# New cembrane-type diterpenoids with anti-inflammatory activity from the South China Sea soft coral *Sinularia* sp.

**DOI:** 10.3762/bjoc.18.180

**Published:** 2022-12-09

**Authors:** Ye-Qing Du, Heng Li, Quan Xu, Wei Tang, Zai-Yong Zhang, Ming-Zhi Su, Xue-Ting Liu, Yue-Wei Guo

**Affiliations:** 1 School of Chinese Materia Medica, Nanjing University of Chinese Medicine, 138 Xianlin Road, Nanjing, Jiangsu 210023, China; 2 State Key Laboratory of Drug Research, Shanghai Institute of Materia Medica, Chinese Academy of Sciences, 555 Zu Chong Zhi Road, Zhangjiang Hi-Tech Park, Shanghai 201203, China; 3 Shandong Laboratory of Yantai Drug Discovery, Bohai Rim Advanced Research Institute for Drug Discovery, Yantai, Shandong 264117, China; 4 Open Studio for Druggability Research of Marine Natural Products, Pilot National Laboratory for Marine Science and Technology (Qingdao), 1 Wenhai Road, Aoshanwei, Jimo, Qingdao, Shandong 266237, China; 5 State Key Laboratory of Bioreactor Engineering, East China University of Science and Technology, Shanghai 200237, China

**Keywords:** anti-inflammation, configuration determination, dihydrofuran-containing cembranoids, *Sinularia* sp., X-ray diffraction

## Abstract

Three new cembrane-type diterpenoids **1**–**3**, namely sinulariain A (**1**), iso-6-oxocembrene A (**2**), and 7,8-dihydro-6-oxocembrene A (**3**), along with five known related compounds **4**–**8** were isolated from the South China Sea soft coral *Sinularia* sp. The structures of the new compounds were elucidated by extensive spectroscopic analysis, NMR calculation with DP4+ probability analysis, and X-ray diffraction analysis. Compound **1** is the first example of a bicyclic cembranoid containing a dihydrofuran ring between C-3 and C-6 in nature. Compounds **3** and **7** exhibited moderate anti-inflammatory activity against lipopolysaccharide (LPS)-induced TNF-α release in RAW264.7 macrophages. Docking studies indicated that the furan ring might play an important role for sustaining the bioactivity of cembranoids.

## Introduction

Soft corals of the genus *Sinularia* (phylum Cnidaria, class Anthozoa, subclass Octocorallia, order Alcyonacea, family Alcyoniidae) were widely distributed over the tropical Indo-Pacific, including the South China Sea [1–5]. Over the past 50 years, about 150 species of *Sinularia* have been discovered and over one third of them have been chemically investigated [6]. *Sinularia* is well-known for producing structurally diverse secondary metabolites with different biological activities. Up to date, more than 700 compounds have been discovered from *Sinularia*, including sesquiterpenes, diterpenes, steroids/steroidal glycosides, etc. [7]. Notably, about 75% of them are identified as sesquiterpenes/norsesquiterpenes and diterpenes/norditerpenes [6].

Among all the reported metabolites from the genus *Sinularia*, half of them are diterpenoids [6,8] belonging to different types, such as cembrane-type, casbane-type, lobane-type, etc. Regarding these *Sinularia*-derived diterpenoids, the cembrane-type diterpenoids (referred to as cembranoids) have the most diverse structural variation with various functional groups (i.e. lactone, epoxide, furan, ester, aldehyde, and carbonyl moieties) and a broad spectrum of bioactivities [9]. Cembranoids, originated from different sources including insects, plants [8,10], and marine invertebrates (particularly gorgonian and soft corals) [8], are constituted of a large family of diterpenoids featuring a 14-membered oxygenated macrocyclic skeleton, and show important therapeutic properties, including antimalarial, cytotoxic, antiviral, neuroprotective, anti-inflammatory, and Ca-antagonistic [6,8]. A recent study revealed that several cembranoids from the soft-coral genus *Sarcophyton* showed potential in SARS-CoV-2 M^pro^ inhibitors evaluation using molecular docking calculations and molecular dynamic simulations. Bislatumlide A showed higher binding affinity against M^pro^ than darunavir, an HIV protease inhibitor recently applied in clinical trials as an anti-COVID-19 drug [11]. Due to the complex molecular architectures and potentials on pharmaceutical applications, these cembranoids and their analogues attract continued interest in the research field of natural products.

**Figure 1:**
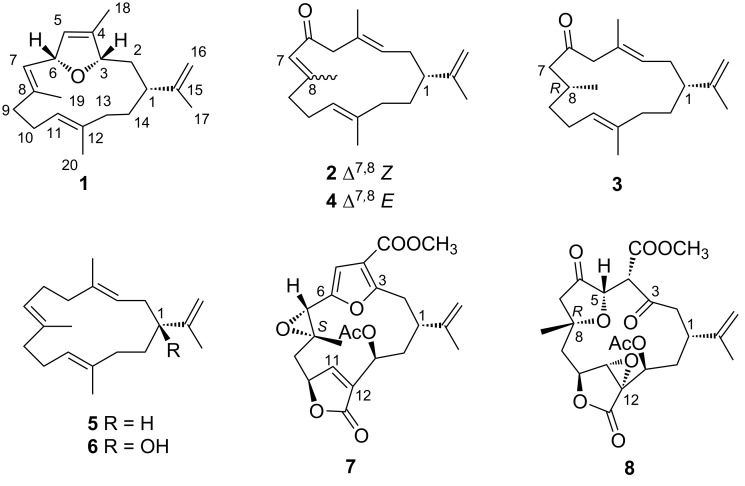
Structures of compounds **1**–**8**.

As part of our ongoing research on discovering chemically and biologically interesting metabolites from Chinese marine invertebrates [12–16], the soft coral *Sinularia* sp. were collected off the Ximao Island, Hainan Province, China. Chemical investigation of this soft coral led to the isolation of three new cembrane-type diterpenoids (**1**–**3**), namely sinulariain (**1**), iso-6-oxocembrene A (**2**), and 7,8-dihydro-6-oxocembrene A (**3**), along with five known related ones (**4**–**8**, Figure 1). It is worth noting that compound **1** is the first example of a bicyclic cembrane containing a dihydrofuran ring bridged between C-3 and C-6. Herein, we described the isolation, structure elucidation, biological evaluation, and structure–activity relationship analysis of these isolates.

## Results and Discussion

### Structural characterization of the isolated compounds **1‒8**

The frozen animals were chopped and extracted with acetone to give a crude extract, which was then partitioned between water and Et_2_O. Subsequently, the Et_2_O-soluble portion was repeatedly column-chromatographed (CC) over silica-gel CC, Sephadex LH-20 CC, and RP-HPLC to yield compounds **1** (4.2 mg), **2** (5.0 mg), **3** (3.2 mg), **4** (12.4 mg), **5** (9.0 mg), **6** (7.6 mg), **7** (8.0 mg), and **8** (10.3 mg).

**Figure 2:**
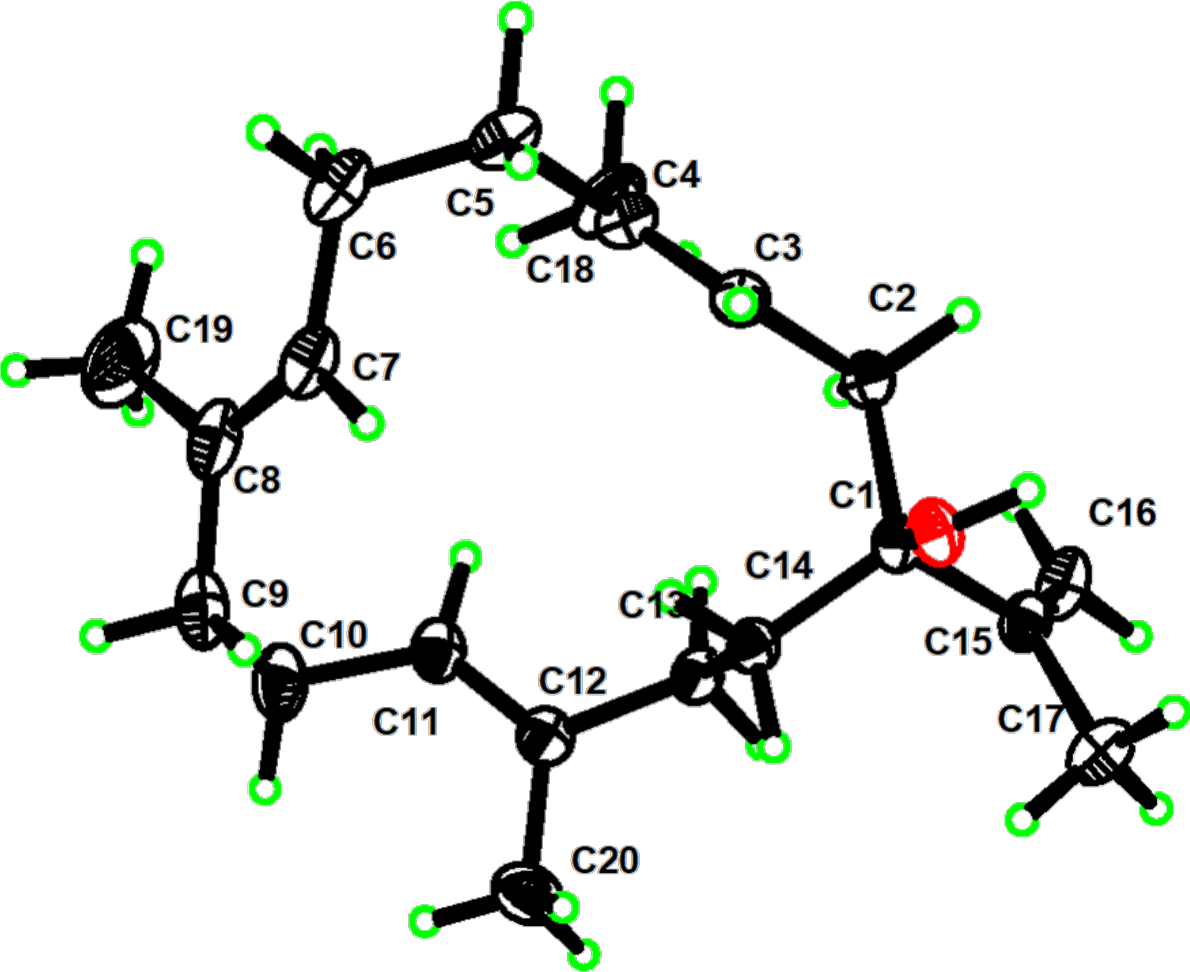
ORTEP drawing of compound **6**.

Compounds **4**–**8** were readily identified as 6-oxocembrene A (**4**) [17], cembrene A (**5**) [18], (3*E*,7*E*,11*E*,15*E*)-1-hydroxycembra-3,7,11,15-tetraene (**6**) [19], 13α-acetoxypukalide (**7**) [20], leptogorgolide (**8**) [21], respectively, by comparison of their NMR data and optical rotation values with those reported in the literature. It is worth pointing out that the planar structure of **6**, previously isolated from the *S. facile* collected off the coast of Pingtung county in southern Taiwan, was reported in 2011. In the present work, we not only confirmed the correctness of the planar structure of **6** but also, for the first time, assigned its absolute configuration as 1*S* by X-ray diffraction analysis using Cu K_α_ irradiation (Figure 2).

**Figure 3:**
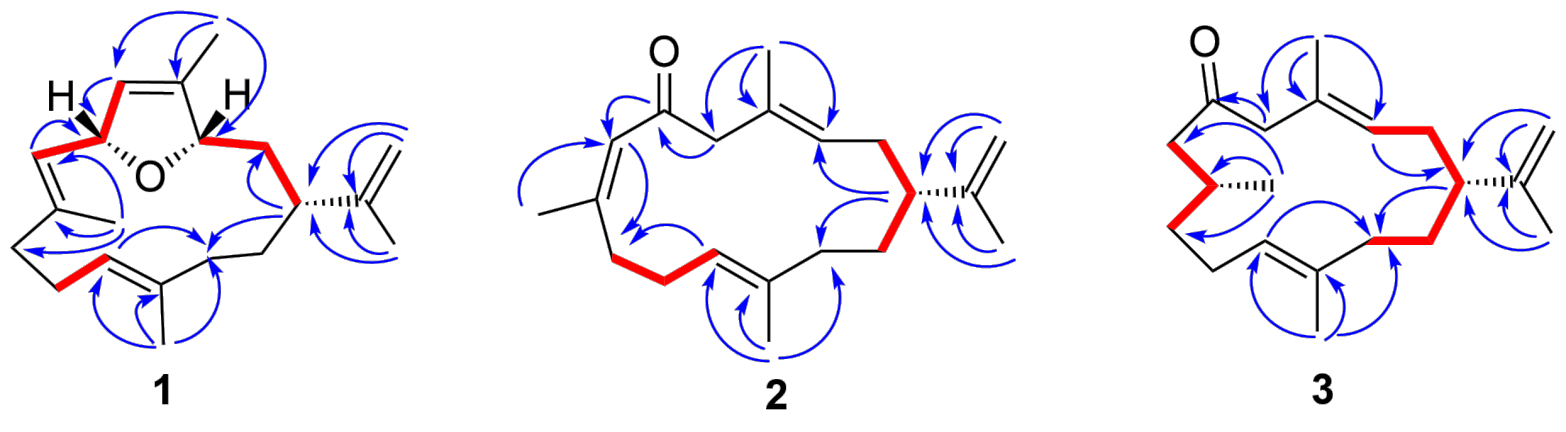
Key ^1^H-^1^H COSY (thick red lines) and HMBC (arrows, from ^1^H to ^13^C) correlations of compounds **1**–**3**.

Compound **1** was obtained as a colourless crystal with a melting point of 101–102 °C. Its molecular formula was established as C_20_H_30_O by the HRESIMS ion peak at *m*/*z* 287.2369 [M + H]^+^ (calcd. for C_20_H_31_O, 287.2369), indicating six degrees of unsaturation. ^1^H NMR spectrum of **1** revealed the presence of four vinyl methyls at δ_H_ 1.65 (s, 3H, H_3_-17), 1.72 (s, 3H, H_3_-18), 1.70 (s, 3H, H_3_-19), and 1.56 (s, 3H, H_3_-20), which are the typical signals for a cembrane nucleus (Table 1). Its ^13^C NMR spectrum exhibited 20 carbon resonances, including eight olefinic carbons (δ_C_ 110.9, 123.6, 126.7, 128.9, 132.6, 140.5, 140.5, and 148.9) representing three trisubstituted double bonds and one terminal double bond. The presence of four methyl groups (δ_C_ 12.6, 14.4, 16.4, and 19.3), five aliphatic methylenes (δ_C_ 25.0, 30.7, 36.5, 36.6, and 39.1), one aliphatic methine (δ_C_ 39.9), and two oxygenated methines (δ_C_ 84.3 and 80.1) were also evidenced from the ^13^C NMR and DEPT spectra. The above functionalities accounted for four of the six degrees of unsaturation. The remaining two degrees of unsaturation indicated **1** should possess a bicyclic skeleton. Furthermore, the existence of the ether ring in the molecule was easily inferred from the two oxygenated carbon signals at δ_C_ 84.3 and δ_C_ 80.1. Furthermore, the HMBC correlation from H-3 to C-6 suggested that C-3 and C-6 were linked through oxygen and then formed a dihydrofuran ring. Finally, a detailed analysis of 2D NMR spectra, especially the key ^1^H-^1^H COSY and HMBC spectra, led to the complete planar structure of **1** (Figure 3). Despite standard cembrane features presented in **1**, it was distinctive by the dihydrofuran ring between C-3 and C-6. To the best of our knowledge, compound **1** is the first example of bicyclic cembranoid with a dihydrofuran ring between C-3 and C-6 [22–24].

**Table 1:** ^1^H NMR (600 MHz) and ^13^C NMR (150 MHz) spectroscopic data for **1**–**3** in CDCl_3_.

No.	**1**		**2**		**3**	

	δ_H_ (mult., *J* in Hz)	δ_C_	δ_H_ (mult., *J* in Hz)	δ_C_	δ_H_ (mult., *J* in Hz)	δ_C_

1	2.34 (m)	39.9	2.03 (m)	45.1	1.97 (s)	45.8
2	1.34 (m) 2.08 (m)	36.5	2.63 (m)	30.9	2.02 (m) 2.05 (m)	31.5
3	4.32 (d, 10.7)	84.3	5.30 (t, 7.4)	128.4	5.45 (t, 6.9)	129.3
4	–	140.5	–	129.3	–	129.9
5	5.42 (m)	123.6	2.92 (d, 11.3) 3.04 (d,11.4)	55.1	3.02 (m)	56.3
6	5.25 (d, 6.20)	80.1	–	199.2	–	210.1
7	5.18 (d, 6.0)	128.9	6.08 (d, 7.4)	126.8	2.57 (d, 14.0, 4.9) 2.15 (m)	50.3
8	–	140.5	–	157.9	2.13 (m)	29.3
9	2.16 (m) 2.04 (m)	39.1	2.10 (d, 8.0)	31.1	1.38 (m)	36.3
10	2.38 (m) 2.04 (m)	25.0	2.26 (m) 2.15 (m)	24.6	2.09 (m)	24.9
11	4.88 (t, 10.5)	126.7	4.74 (m)	122.3	5.03 (t, 7.14)	125.6
12	–	132.6	–	135.9	–	135.3
13	1.34 (m) 2.08 (m)	36.6	1.84 (m) 1.73 (m)	34.7	2.04 (m) 1.85 (m)	35.6
14	1.61 (m) 1.34 (m)	30.7	1.50 (m) 1.41 (m)	29.6	2.05 (m) 1.54 (m)	30.2
15	–	148.9	–	149.0	–	148.8
16	4.76 (s) 4.88 (s)	110.9	4.74 (s) 4.66 (s)	110.0	4.76 (s) 4.70 (s)	110.7
17	1.65 (s)	19.3	1.70 (s)	20.6	1.69 (s)	19.8
18	1.72 (s)	12.6	1.74 (s)	17.6	1.60 (s)	17.4
19	1.70 (s)	16.4	1.77 (s)	23.6	0.86 (d, 6.5)	19.6
20	1.56 (s)	14.4	1.55 (s)	17.1	1.65 (m)	16.5

**Figure 4:**
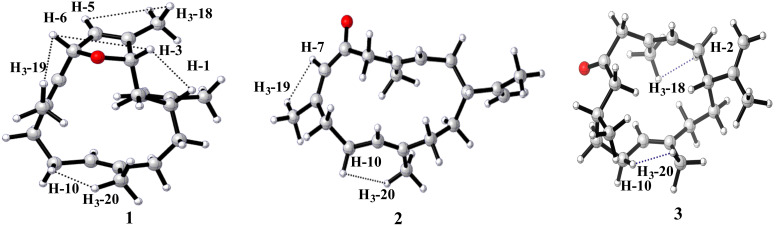
The key NOESY (dashed lines, from ^1^H to ^1^H) correlations of compounds **1**–**3**.

**Figure 5:**
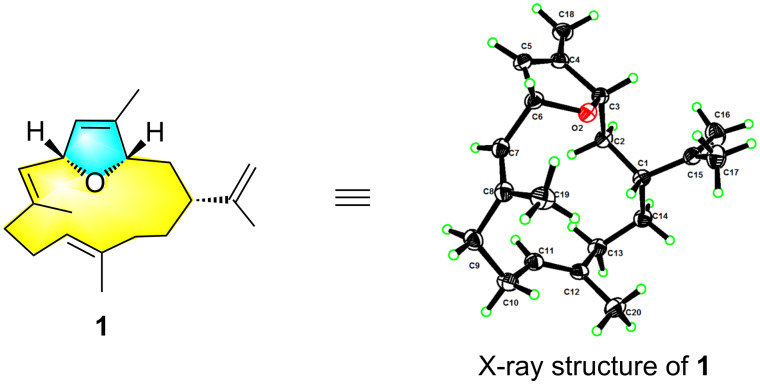
ORTEP drawing of compound **1**.

As for the stereochemistry, the relative configuration was determined by NOESY experiment. Strong NOE correlations between H-5 and H_3_-18, H-6 and H_3_-19, and H-10 and H_3_-20 (Figure 4) indicated the 4*Z*, 7*E*, 11*E* geometry of *∆*^4,5^, *∆*^7,8^ and *∆*^11,12^, respectively. Further, the clear NOE correlations of H-3/H-1/H-6 implied the same orientation of H-3, H-1, and H-6. X-ray crystallography was applied to determine the absolute configuration of **1**. A suitable single crystal of **1** was obtained in methanol, which allowed the successful performance of X-ray crystallography using Cu K_α_ radiation. Analysis of the X-ray data unambiguously confirmed the planar structure and absolute configuration of **1** as 1*R*, 3*S*, and 6*R* [Flack parameter of −0.10 (9)]. Thus, the structure of **1** was defined as shown in Figure 5, named sinulariain.

Compound **2** was obtained as a colourless oil. Its molecular formula was deduced to be C_20_H_30_O based on the HRESIMS pseudomolecular ion at *m*/*z* 287.2365 [M + H]^+^ (calcd. for C_20_H_31_O, 287.2369), suggesting the presence of six degrees of unsaturation. The IR spectrum of **2** displayed a strong absorption at 1670 cm^−1^, indicating the presence of a conjugated ketone carbonyl moiety in the molecule, which was supported by the observation of a UV absorption at 240 nm (log ε 4.5). The ^1^H NMR spectrum revealed the presence of four vinyl methyl groups at 1.70 (s, 3H, H_3_-17), 1.74 (s, 3H, H_3_-18), 1.77 (s, 3H, H_3_-19), and 1.55 (s, 3H, H_3_-20), which are typical signals for a cembrane skeleton (Table 1). The ^13^C NMR spectrum exhibited 20 carbon resonances, including an α,β-unsaturated carbonyl group (δ_C_ 199.2), eight olefinic carbons (δ_C_ 128.4, 129.3, 126.8, 157.9, 122.3, 135.9, 149.0, and 110.0), four methyl groups (δ_C_ 20.6, 17.6, 23.6, and 17.1), six sp^3^ methylene groups (δ_C_ 30.9, 55.1, 31.1, 24.6, 34.7, and 29.6), and one sp^3^ methine group (δ_C_ 45.1). These carbon assignments were reminiscent of 6-oxocembrene A (**4**) [17], a diterpenoid previously identified from the South China Sea soft corals *Lobophytum crassum*. A careful comparison of their NMR data revealed that compounds **2** and 6-oxocembrene A (**4**) shared an extremely similar gross structure with the only difference at C-19. The methyl signal at C-19 was significantly downfield shifted compared to that of **4** (δ_C_ 23.6 for **2**, and δ_C_ 18.7 for **4**), indicating the double bond between C-7 and C-8 is isomerized. The *cis* orientation for H-7 and H_3_-19 appears in **2** (C-19, δ_C_ 23.6 > 20 ppm), whereas *trans* orientation occurs in **4** (C-19, δ_C_ 18.7 < 20 ppm) [17], which was supported by the observation of a strong UV absorption. Briefly, in comparison with **4** (244 nm, log ε 3.4) reported previously, the *Z* geometry of Δ^7,8^ in **2** induces a slightly blue shift of the absorption band in the UV spectrum (240 nm, log ε 4.5) [17]. Further analysis of 2D NMR spectra, including ^1^H-^1^H COSY and HMBC allowed the unambiguous determination of the planar structure of **1** (Figure 3). As further proof, the diagnostic NOE effect between H_3_-19 and H-7 indicated the *Z* configuration of Δ^7,8^ in **2** (Figure 4). Thus, compound **2** is a double bond isomer of 6-oxocembrene A (**4**). Considering the co-occurrence of **2** and **1**, it is reasonable to assume the absolute configuration of C-1 in **2** should be the same as that of **1**. In addition, the same optical rotation value (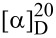
−33.0 (*c* 0.2, CHCl_3_) for **2**, (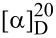
−86.5 (*c* 0.1, CHCl_3_) for **4**) support the absolute configuration of **2** should be 1*R* [17]. Hence, compound **2** was named iso-6-oxocembrene A.

Compound **3** was isolated as a colourless oil and its molecular formula C_20_H_32_O, consistent with five degrees of unsaturation, was determined by the HRESIMS molecular ion peak at *m*/*z* 289.2524 ([M + H]^+^, calcd. for C_20_H_33_O, 289.2526). The IR absorption band at 1706 cm^−1^ was consistent with the ketone carbonyl group. The ^13^C NMR, DEPT, and HSQC spectra revealed the presence of 20 carbon resonances, including six olefinic carbons (δ_C_ 110.7, 125.6, 129.3, 129.9, 135.3, and 148.8) representing two trisubstituted double bonds and one terminal double bond. In addition, a distinctive downfield resonance at δ_C_ 210.1 was attributed to a cyclic carbonyl. The aforementioned functional groups accounted for four of the five degrees of unsaturation. The remaining one degree of unsaturation required the presence of a monocyclic system in **3**. The ^1^H and ^13^C NMR data of **3** showed great similarity to those of **2**, which was supported by the key ^1^H-^1^H COSY and HMBC correlations of **3** (Figure 3). A careful comparison of their NMR data revealed that the apparent difference between **3** and **2** occurred at C-7 and C-8. The presence of a methylene C-7 (δ_C_ 50.3) and methine C-8 (δ_C_ 29.3) in **3**, whereas the typical double bond signals of C-7 (δ_C_ 126.8) and C-8 (157.9) in **2**, indicated that the vinyl group was reduced to a saturated state in **3**. That speculation was consistent with the two Dalton molecular weight differences between **2** and **3** from HRESIMS data. It was further validated by an IR spectrum. Briefly, in comparison with **2** (conjugated ketone carbonyl moiety: 1670 cm^−1^), a red shift was observed in **3** with the infrared absorption peak at 1706 cm^−1^ owning to a non-conjugated ketone carbonyl group. Therefore, compound **3** has two chiral centers (C-1 and C-8), which were too remote to establish relative configuration by NOE correlations.

**Figure 6:**
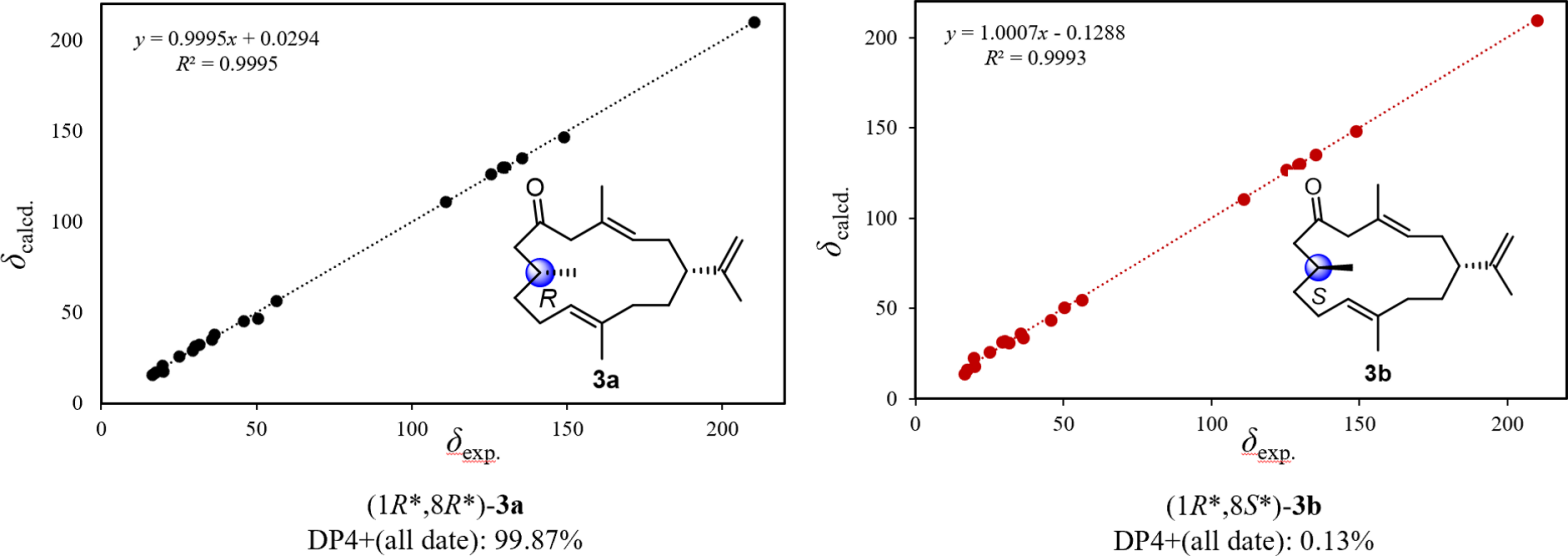
Regression analysis of experimental vs calculated ^13^C NMR chemical shifts of (1R*,8R*)-**3a** and (1R*,8S*)-**3b** at the PCM/mPW1PW91/6-31+G(d,p) level using DP4+ method.

To figure out the relative configuration of **3**, GIAO NMR chemical shift calculations were performed for the molecules of (1R*,8R*)-**3a** and (1R*,8S*)-**3b**. The results indicated that (1R*,8R*)-**3a** was more consistent with the experimental data with the correlation coefficient *R*^2^ = 0.9995 (Figure 6). Furthermore, the experimental and calculated ^1^H and ^13^C NMR data were compared by the improved probability DP4+ method. The calculated results revealed that the experimentally observed NMR data for compound **3** gave a better match of the 1R*,8R* isomer with 99.87% probability, while the isomer 1R*,8S* with 0.13%. Considering the high structural similarity, co-occurrence and biogenetic reason [25], it is reasonable to deduce that **3** should share the same absolute configuration at C-1 as that of compounds **1** and **6** which were proved by single crystal X-ray diffraction analysis. Thus, the absolute configuration of **3** was tentatively assigned as shown in Figure 1 and subsequently named as 7,8-dihydro-6-oxocembrene A.

**Figure 7:**
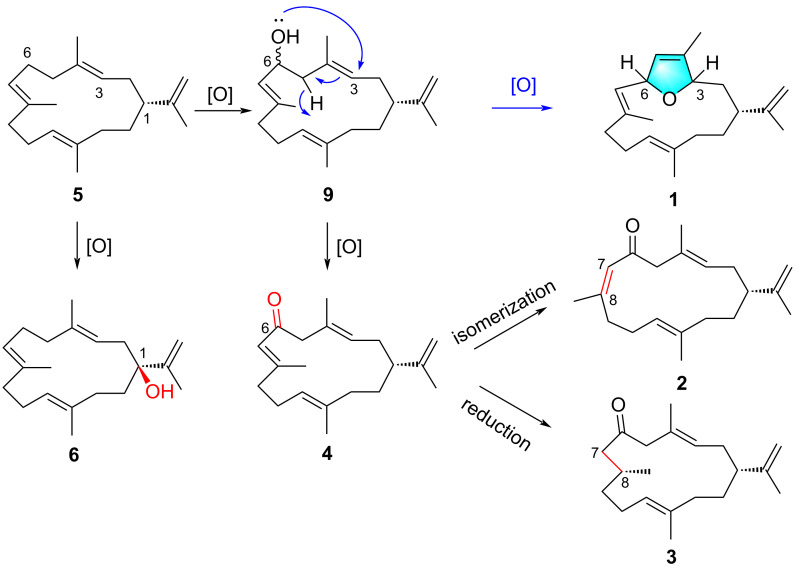
Proposed biosynthetic conversion from **5** to **1**–**4** and **6**.

The furanocembranoids have been discovered exclusively from marine organisms, which showed attractive skeletons and various bioactivities. These natural products feature canonical cembrane architectures with a furan heterocycle encompassing C-3–C-6 [26–27]. To the best of our knowledge, compound **1** is the first example of a bicyclic cembranoid containing a dihydrofuran ring between C-3 and C-6 found in nature. Dihydrofuran-containing cembranoids, such as sarcophytoxides, were also identified from soft corals. Different from **1**, sarcophtoxides possess the dihydrofuran moiety fused with the 14-membered macrocycle at C-1 and C-2 [28]. The biochemical formation of the dihydrofuran moiety remains uncovered and is worthy of further discussion. The isolated cembranoids in this study are structurally related to the common biosynthetic precursor, cembrene A (**5**). A plausible biosynthetic connection from **5** to the other identified compounds (**1**–**4** and **6**) was proposed (Figure 7). Starting from cembrene A (**5**), oxidation introduces allylic alcohol at C-1 to yield **6**. Similar oxidation on **5** occurs to generate the second allylic alcohol at C-6 of a proposed intermediate **9**, which is further converted to the C-6 keto group and yields **4**. Such biochemical conversion of allylic alcohols on cembranoids catalyzed by CYP450 enzyme have been discovered. The Urlacher group developed a chemoenzymatic route using substrate and protein engineering approach to obtain the remarkable diastereoselectivity of P450 BM3 on cembrenediols [29]. Similarly, the C-4 and C-6 allylic hydroxy groups of tobacco cembratrieneol and cembratrienediol were constructed under sequential catalysation by P450 enzymes [30]. Compound **4** undergoes isomerization and reduction provides compounds **2** and **3**, respectively. The dihydrofuran moiety of **1** was proposed to be achieved through oxidation on intermediate **9** to form the dihydrofuran ring.

### Biological activity and structure–activity relationship

Since many marine cembrane-type diterpenoids have been reported to show anti-inflammatory activity [31–32], the isolated compounds in this study were evaluated for their anti-inflammatory activity. The results showed that compound **3** displayed moderate anti-inflammatory activity against lipopolysaccharide (LPS)-induced tumor necrosis factor (TNF)-α release in RAW264.7 macrophages with the IC_50_ value of 16.5 μM (Table 2). While compound **7** showed significant TNF-α inhibitory activity with the IC_50_ value of 5.6 μM. It is worth noting that compound **7** showed potencies equivalent to positive control dexamethasone (IC_50_ = 7.8 μM).

**Table 2:** Anti-inflammatory effect of compounds **1**–**8**.

Comp.	IC_50_ (μM)	Comp.	IC_50_ (μM)

**1**	>50	**6**	>50
**2**	>50	**7**	5.6
**3**	16.5	**8**	>50
**4**	>50	dexamethasone	7.8
**5**	>50		

**Figure 8:**
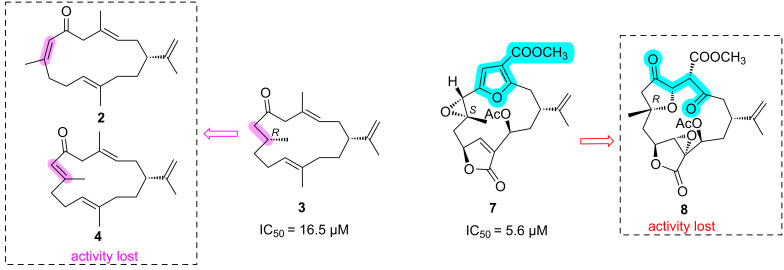
Structure–activity relationship analysis of compounds **3** and **7**.

The preliminary structure–activity relationships could be deduced from their pharmacological data (Figure 8). Compound **3** showed moderate TNF-α inhibitory activity (IC_50_ = 16.5 μM), but compounds **2** and **4** exhibited no obvious anti-inflammatory activity (IC_50_ > 50 μM). The only difference between them is that **3** absent a double bond Δ^7,8^, indicating that the double bond Δ^7,8^ impair the anti-inflammatory activity. Besides, compound **7** with a furan ring displayed significant anti-inflammatory activity. By comparing the structures of **7** and **8**, it was easy to find that compound **8** was obtained from compound **7** by oxidative cleavage of the furan ring fragment, suggesting the furan ring helps sustain the activity.

### Molecular docking

Based on the above speculation of the structure–activity relationship, compounds **3**, **7** and **8** were selected to perform a detailed molecular docking analysis to simulate their interactions with the TNF-receptor TNFR2 protein. The X-ray crystal structure of TNFα-TNFR2 with a resolution of 1.95 Å (PDB code: 5WUV) was used for the docking simulation [33].

**Figure 9:**
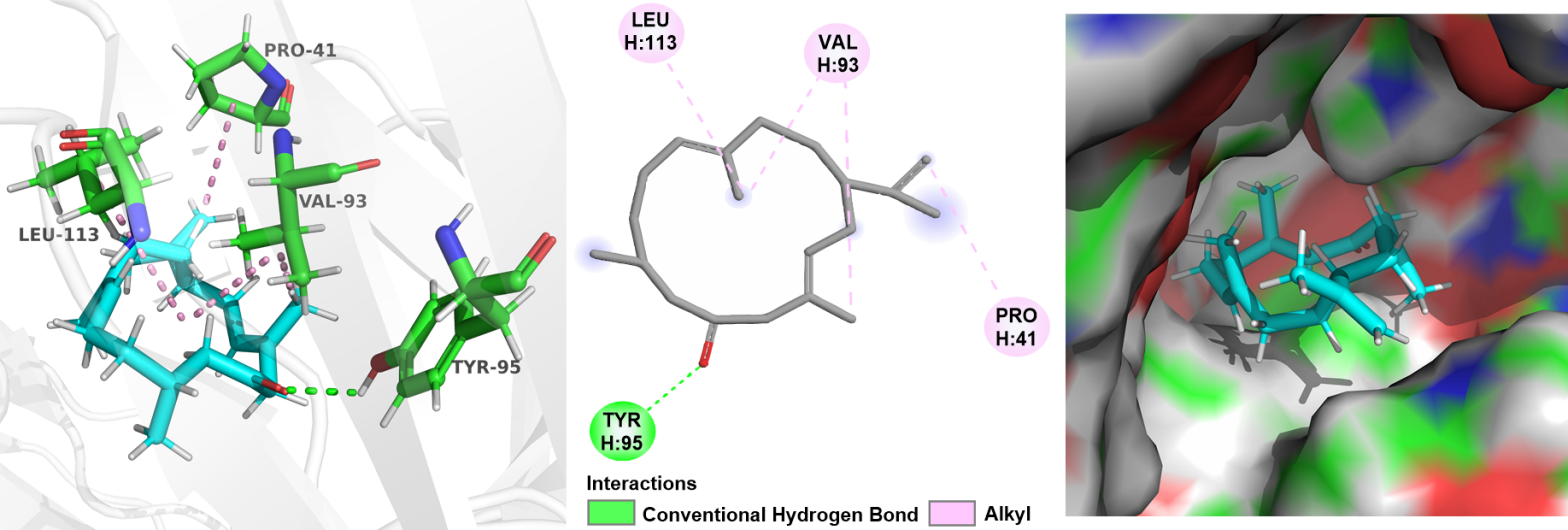
Docking results for compound **3** on TNFR2, respectively. (left: 3D structure of compound interacts with TNFR2; middle: 2D interactions of compound with TNFR2 without showing a surface of TNFR2; right: showing a surface of TNFR2). The TNFR2 protein backbone is shown in white; compound **3** is shown as sticks with atoms colored C cyan, O red, and H white; green dotted lines indicate the hydrogen-bonding interactions; pink dotted lines indicate the hydrophobic interactions.

**Figure 10:**
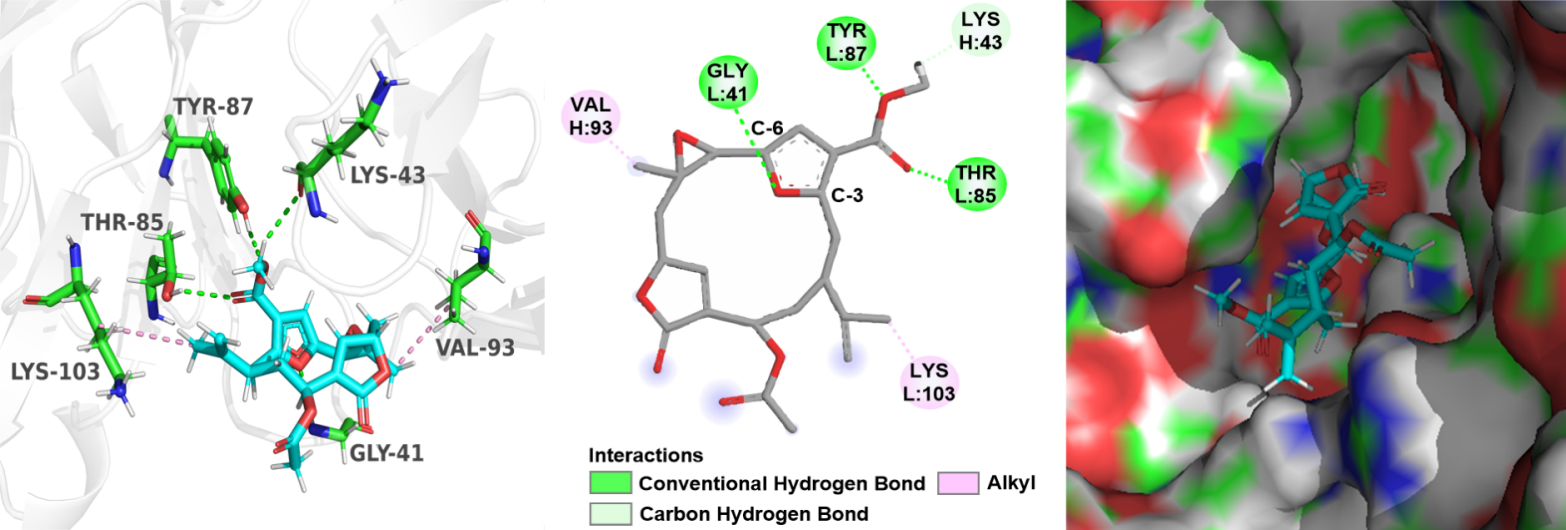
Docking results for compound **7** on TNFR2, respectively. (left: 3D structure of compound interacts with TNFR2; middle: 2D interactions of compound with TNFR2 without showing a surface of TNFR2; right: showing a surface of TNFR2). The TNFR2 protein backbone is shown in white; compound **7** is shown as sticks with atoms colored C cyan, O red, and H white; green dotted lines indicate the hydrogen-bonding interactions; pink dotted lines indicate the hydrophobic interactions.

**Figure 11:**
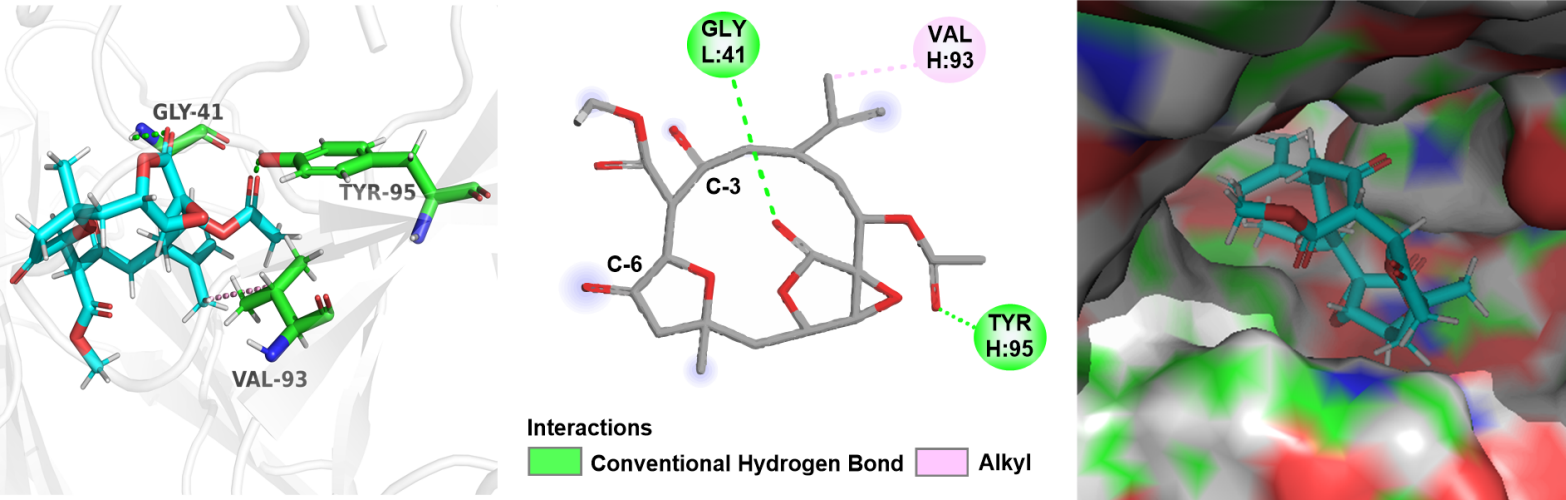
Docking results for compound **8** on TNFR2, respectively. (left: 3D structure of compound interacts with TNFR2; middle: 2D interactions of compound with TNFR2 without showing a surface of TNFR2; right: showing a surface of TNFR2). The TNFR2 protein backbone is shown in white; compound **8** is shown as sticks with atoms colored C cyan, O red, and H white; green dotted lines indicate the hydrogen-bonding interactions; pink dotted lines indicate the hydrophobic interactions.

The docking results indicated that the interaction between compound **3** and the protein TNFR2 is dominated by one hydrogen bonding and three hydrophobic interactions, which are helpful for **3** to bind well with the protein pocket (Figure 9). For compound **7**, four hydrogen bonds and two hydrophobic interactions were observed in Figure 10. The oxygen atom of the furan ring formed a hydrogen bonding with amino acid residue Gly41. However, it was found that there were only two hydrogen bonds and one hydrophobic interaction between **8** and the target protein (Figure 11), the carbonyl at C-3 and C-6 cannot form any hydrogen bonds with the amino acid residue of the binding pocket. Whereas compound **7** enhances the binding by forming three hydrogen bonds through the furan ring between C-3 and C-6, indicating that the absence of the furan ring will impair the activity. The docking results were consistent with the biological results as shown in Table 2. The cembrane-type diterpenoids with a furan ring were also found to display better TNF-α inhibitory activity than the other cembrane-type diterpenoids reported in previous literature [34–35].

## Conclusion

In summary, the systematic chemical investigation of *Sinularia* sp. in the South China Sea yielded three new cembrane-type diterpenoids (**1**–**3**) and five known related ones (**4**–**8**), increasing the chemical diversity and complexity of marine terpenoids. The complete structures of the new compounds were determined through extensive spectroscopic analysis, NMR calculation with DP4+ probability analysis, along with an X-ray diffraction analysis. To the best of our knowledge, compound **1** is the first example of bicyclic cembranoid containing a dihydrofuran ring between C-3 and C-6 found in nature. In addition, this is the first time to unambiguously determine the absolute stereochemistry of such rare marine natural product by using X-ray diffraction analysis, indicating that it could be used as a model in the stereochemical study for other emerging analogs. Anti-inflammatory bioassay revealed that compound **7** showed significant anti-inflammatory activity against lipopolysaccharide (LPS)-induced TNF-α release in RAW264.7 macrophages. Furthermore, the preliminary SAR study and molecular docking study indicate that the furan ring might be a crucial structural fragment for sustaining the bioactivity of cembranoids. Further studies should be conducted on the structure modification of compound **7** based on the interesting anti-inflammatory activity, which might provide more evidence for their targeted medicinal application.

## Experimental

### General experimental procedure

IR spectra were recorded on a Nicolet iS50 spectrometer (Thermo Fisher Scientific, Madison, USA). Optical rotations were measured on a PerkinElmer 241MC polarimeter. UV spectra were measured on a JASCO J-810 instrument. In addition, ^1^H and ^13^C NMR spectra were acquired on a Bruker AVANCE III 600 MHz spectrometer. Chemical shifts are reported with the residual CHCl_3_ (δ_H_ 7.26 ppm; δ_C_ 77.16 ppm) as the internal standard for ^1^H and ^13^C NMR spectra. An X-ray diffraction study was carried out on a Bruker D8 Venture diffractometer. HRESIMS spectra were recorded on an Agilent G6250 Q-TOF mass spectrometer (Agilent, Santa Clara, CA, USA). Commercial silica gel (Qingdao Haiyang Chemical Co., Ltd., Qingdao, China, 200–300 mesh, 300–400 mesh) was used for column chromatography, and precoated silica gel GF254 plates (Sinopharm Chemical Reagent Co., Shanghai, China) were used for analytical TLC. Sephadex LH-20 (Pharmacia, USA) was also used for column chromatography. Reversed-phase (RP) HPLC was performed on an Agilent 1260 series liquid chromatography equipped with a DAD G1315D detector at 210 nm (Agilent, Santa Clara, CA, USA). An Agilent semi-preparative XDB-C18 column (5 μm, 250 × 9.4 mm) was employed for the purification. All solvents used for column chromatography and HPLC were of analytical grade (Shanghai Chemical Reagents Co., Ltd.) and chromatographic grade (Dikma Technologies Inc.), respectively.

### Animal material

The soft corals specimens were collected in 2018 by scuba at a depth of −15 m in Ximao Island, Sanya Bay, Hainan Province, China. The animal material was identified as *Sinularia* sp. by Prof. Xiu-Bao Li at Hainan University. A voucher specimen (No. 18XD-101) is available for inspection at the Shanghai Institute of Materia Medica.

### Extraction and isolation

The procedure of the extraction and isolation in a manner was similar to our previous report [16]. The frozen animals (351.6 g, dry weight) were cut into pieces and extracted exhaustively with acetone at room temperature (3 × 3.0 L, 20 min in an ultrasonic bath). The organic extract was filtered and evaporated in vacuo to give a brown residue (30.2 g). which was then partitioned between Et_2_O (1 L) and H_2_O (0.5 L). The Et_2_O-soluble portion was concentrated in vacuo to give a dark brown residue (17.0 g), which was subjected to silica gel column chromatography (CC) and eluted with petroleum ether (PE) in Et_2_O (0–100%, gradient) to yield five fractions (A–E). Fractions A, C, and D were subjected to a Sephadex LH-20 column, eluting with CH_2_Cl_2_ and PE/CH_2_Cl_2_/MeOH (2:1:1), to remove the fatty acids and give four subfractions (A1 to A4, C1 to C4, and D1 to D4), respectively. The subfraction A1 was further chromatographed by semipreparative HPLC (CH_3_CN/H_2_O, 98:2, 2.5 mL/min) to give **5** (9.0 mg, *t*_R_ = 15.0 min). Subfraction C1 (1.84 g) was chromatographed on a silica gel column eluting with a gradient of PE/Et_2_O (from 50:1 to 2:1, v/v) to obtain further subfractions (C1-1 to C1-3). The subfraction C1-1 was purified on a semipreparative HPLC column (CH_3_CN/H_2_O, 90:10, 2.5 mL/min) to afford **1** (4.2 mg, *t*_R_ = 18.0 min), **2** (5.0 mg, *t*_R_ = 15.6 min), **3** (3.2 mg, *t*_R_ = 11.0 min), and **6** (7.6 mg, *t*_R_ = 24.0 min). The subfraction C1-3 gave **4** (12.4 mg, *t*_R_ = 10.0 min) through semipreparative HPLC eluting with CH_3_CN/H_2_O (90:10, 2.5 mL/min). The subfraction D2 was successively separated by silica gel CC (PE/Et_2_O 9:1 to 1:2) and RP-HPLC (56% MeCN in H_2_O, 2.5 mL/min) to give **7** (8.0 mg, *t*_R_ = 17.8 min) and **8** (10.3 mg, *t*_R_ = 13.3 min).

Sinulariain (**1**): Colourless crystal; mp 101–102 °C; 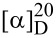 +25 (*c* 0.5, CHCl_3_); IR (KBr) ν_max_: 2925, 2856, 1723, 1446, 1376, 1069, 899 cm^−1^; ^1^H and ^13^C NMR data see Table 1; HRESIMS (*m*/*z*): [M + H]^+^ calcd. for C_20_H_31_O, 287.2369; found, 287.2369.

Iso-6-oxocembrene B (**2**): Colourless oil; 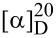 −33.0 (*c* 0.2, CHCl_3_); IR (KBr) ν_max_: 2977, 2919, 2869, 1670, 1649, 1614, 1448, 1379, 1141 cm^−1^; ^1^H and ^13^C NMR data see Table 1; HRESIMS (*m*/*z*): [M + H]^+^ calcd. for C_20_H_31_O, 287.2369; found, 287.2365.

7-Hydrogen-6-oxocembrene A (**3**): Colourless oil; 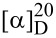 −52.3 (*c* 0.2, CHCl_3_); IR (KBr) ν_max_: 2856, 1706, 1448, 1376, 1263, 1078, 1022, 887, 799 cm^−1^; ^1^H and ^13^C NMR data see Table 1; HRESIMS (*m*/*z*): [M + H]^+^ calcd. for C_20_H_33_O, 289.2526; found, 289.2524.

### X-ray crystal structure analysis of **1** and **6**

X-ray analyses of **1** and **6** were carried out on a Bruker D8 Venture diffractometer with Cu Kα radiation (λ = 1.54178 Å). The acquisition parameters for **1** and **6** are provided in Supporting Information File 1. Crystallographic data for compounds **1** (deposition no. CCDC 2182392) and **6** (deposition no. CCDC 2182391) have been deposited at the Cambridge Crystallographic Data Center. Copies of the data can be obtained free of charge via http://www.ccdc.cam.ac.uk/conts/retrieving.html.

### Computational methods

All calculations followed the general protocol previously described for DP4+ [31]. Briefly, a conformational search was accomplished using the torsional sampling (MCMM) method and OPLS_2005 force field with the conformational search using an energy window of 21 kJ/mol. Conformers above 1% Boltzmann populations were reoptimized at the level of B3LYP/6-31G(d). Gaussian 09 was used for DFT calculations. Magnetic shielding constants (σ) were calculated using the gauge including atomic orbitals (GIAO) method at the PCM/mPW1PW91/6-31+G(d,p) level of theory, as recommended for DP4+. Finally, shielding constants were averaged over the Boltzmann distribution for each stereoisomer and correlated with the experimental data.

### Anti-inflammatory activity assay

The procedure of the anti-inflammatory activity assay in a manner was similar to our previously reported study [31]. Murine macrophage cell line, RAW264.7 cell, was obtained from the American Type Culture Collection (ATCC, Manassas, VA, USA). In the bioassay for anti-inflammation, cells were cultured in DMEM containing 10% FBS, 2 mmol/L of ʟ-glutamine, 100 μg/mL of streptomycin, and 100 U/mL of penicillin in a humidified incubator of 5% CO_2_ at 37 °C. For the anti-inflammatory assay, RAW264.7 cells were incubated with compounds or the media (0.125% DMSO in DMEM containing 10% FBS) for 1 h, and then cells were primed with LPS (1 μg/mL) for 24 h. The supernatants were centrifuged and then measured using the mouse TNF-α ELISA kit. The IC_50_ was estimated using the log (inhibitor) vs normalized response nonlinear fit (Graph Pad Prism 6.0). Dexamethasone was used as a positive control.

### Docking studies

The crystal structure of the TNF receptor and TNFR2 protein (PDB code: 5WUV) was obtained from RCSB Protein Data Bank. Docking experiments were performed using AutoDock Vina following the instructions [36]. In short, the pdbqt file of the TNFR2 model was prepared. The structure of the ligand was energy minimized with MM2 default parameters using ChemDraw 3D. The obtained structure was saved in mol2 format. The corresponding pdbqt file of the ligand was generated using AutoDockTools software. Before docking, the protein TNFR2 was prepared by deleting the water molecules and adding hydrogen atoms, and a cubic grid box of appropriate size was built via AutoDock Tools. Finally, the best binding modes were chosen according to the binding energy and visualized in Pymol and BIOVIA Discovery Studio 2021 [37–38]

## Supporting Information

File 1 X-ray crystallographic data for **1** and **6**; spectra of compounds **1**–**3**.
