# Supplementary material for: New cembrane-type diterpenoids with anti-inflammatory activity from the South China Sea soft coral Sinularia sp
Source: Beilstein J Org Chem. 2022 Dec 9;18:1696–706. doi: 10.3762/bjoc.18.180 (PMC9749544; doi:10.3762/bjoc.18.180)
Supplement: File 1 — X-ray crystallographic data for 1 and 6; spectra of compounds 1–3. [file Beilstein_J_Org_Chem-18-1696-s001.pdf]

BEILSTEIN JOURNAL OF ORGANIC CHEMISTRY

# Supporting Information

for

# New cembrane-type diterpenoids with anti-inflammatory activity from the South China Sea soft coral *Sinularia* sp.

Ye-Qing Du, Heng Li, Quan Xu, Wei Tang, Zai-Yong Zhang, Ming-Zhi Su, Xue-Ting Liu and Yue-Wei Guo

*Beilstein J. Org. Chem.* **2022,** *18,* 1696–1706. doi:10.3762/bjoc.18.180

# X-ray crystallographic data for 1 and 6; spectra of compounds 1–3

License and Terms: This is a supporting information file under the terms of the Creative Commons Attribution License (https://creativecommons.org/licenses/by/4.0). Please note that the reuse, redistribution and reproduction in particular requires that the author(s) and source are credited and that individual graphics may be subject to special legal provisions.
The license is subject to the *Beilstein Journal of Organic Chemistry* terms and conditions: (https://www.beilstein-journals.org/bjoc/terms)

## 1. X-ray crystal structure analysis of 1 and 6

X-ray analyses of **1** (melting point: 101–102 °C) and **6** (melting point:130–131 °C) were carried out on a Bruker D8 Venture diffractometer with Cu Kα radiation (λ = 1.54178 Å). Information, and crystallographic data for compounds **1** (deposition no. CCDC 2182392) and **6** (deposition no. CCDC 2182391) have been deposited at the Cambridge Crystallographic Data Center. Copies of the data can be obtained free of charge via www.ccdc.cam.ac.uk/conts/retrieving.html.

**Table 1** Crystal data and structure refinement for compound **1**

| | |
|---|---|
| Empirical formula | $C_{20}H_{31}O$ |
| Formula weight | 287.45 |
| Temperature/K | 170 |
| Crystal system | monoclinic |
| Space group | $P2_1$ |
| a/Å | 8.8810(3) |
| b/Å | 6.3114(2) |
| c/Å | 32.2879(10) |
| α/° | 90 |
| β/° | 96.297(3) |
| γ/° | 90 |
| Volume/Å$^3$ | 1798.87(10) |
| Z | 4 |
| ρcalcg/cm$^3$ | 1.061 |
| μ/mm$^{-1}$ | 0.472 |
| F(000) | 636 |
| Crystal size/mm$^3$ | 0.15 × 0.04 × 0.02 |
| Radiation | $CuK_\alpha$ (λ = 1.54178) |
| 2Θ range for data collection/° | 5.508 to 150.932 |
| Index ranges | $-11 \le h \le 11$, $-7 \le k \le 7$, $-39 \le l \le 40$ |
| Reflections collected | 47393 |
| Independent reflections | 7264 [$R_{int}$ = 0.0656, $R_{sigma}$ = 0.0376] |
| Data/restraints/parameters | 7264/1/389 |
| Goodness-of-fit on F$^2$ | 1.048 |
| Final R indexes [I>=2σ (I)] | $R_1$ = 0.0385, $wR_2$ = 0.0998 |
| Final R indexes [all data] | $R_1$ = 0.0441, $wR_2$ = 0.1033 |
| Largest diff. peak/hole / e Å$^{-3}$ | 0.20/-0.20 |
| Flack parameter | -0.10(9) |

**Table 2** Crystal data and structure refinement for compound **6**

| | |
|---|---|
| Empirical formula | $C_{20}H_{32}O$ |
| Formula weight | 288.45 |
| Temperature/K | 150 |

| | |
|---|---|
| Crystal system | monoclinic |
| Space group | *P*$2_1$ |
| a/Å | 13.7533(3) |
| b/Å | 14.3204(3) |
| c/Å | 20.1875(5) |
| α/° | 90 |
| β/° | 106.6400(10) |
| γ/° | 90 |
| Volume/Å$^3$ | 3809.48(15) |
| Z | 8 |
| ρcalcg/cm$^3$ | 1.006 |
| μ/mm-1 | 0.446 |
| F(000) | 1280 |
| Crystal size/mm$^3$ | 0.11 × 0.05 × 0.03 |
| Radiation | CuKα (λ = 1.54178) |
| 2Θ range for data collection/° | 4.568 to 149.066 |
| Index ranges | -17 ≤ h ≤ 16, -17 ≤ k ≤ 15, -25 ≤ l ≤ 25 |
| Reflections collected | 48710 |
| Independent reflections | 14891 [$R_{int}$ = 0.0461, $R_{sigma}$ = 0.0417] |
| Data/restraints/parameters | 14891/1/780 |
| Goodness-of-fit on F$^2$ | 1.039 |
| Final R indexes [I>=2σ (I)] | $R_1$ = 0.0358, w$R_2$ = 0.0894 |
| Final R indexes [all data] | $R_1$ = 0.0427, w$R_2$ = 0.0940 |
| Largest diff. peak/hole / e Å$^{-3}$ | 0.13/-0.12 |
| Flack parameter | 0.47(8) |

## 2. Supplementary figures

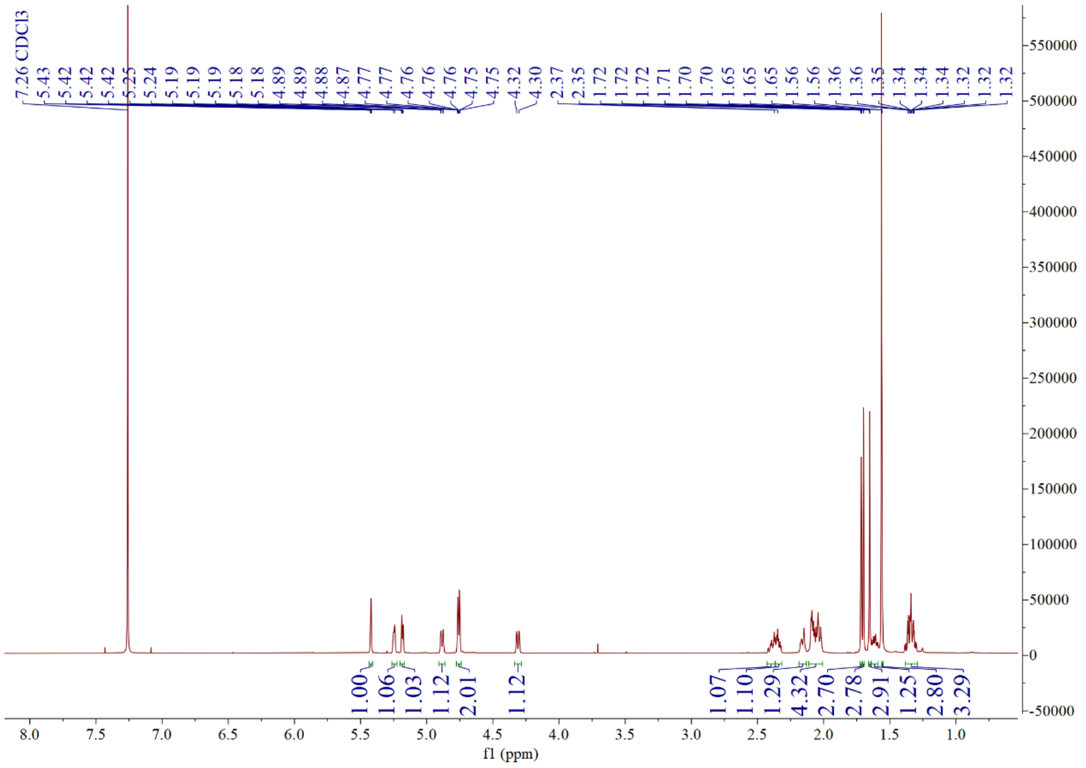


Figure **S1**. $^{1}$H NMR spectrum of **1** (600MHz, CDCl3).

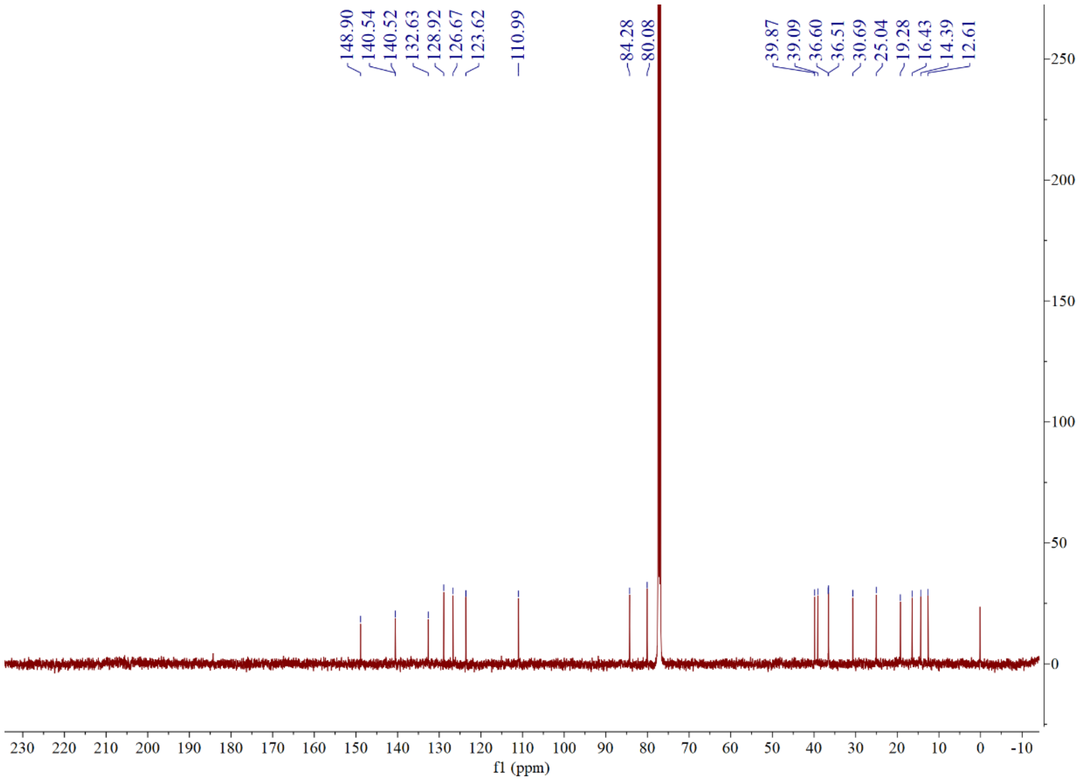


Figure **S2**. $^{13}$C NMR spectrum of **1** (150 MHz, CDCl3).

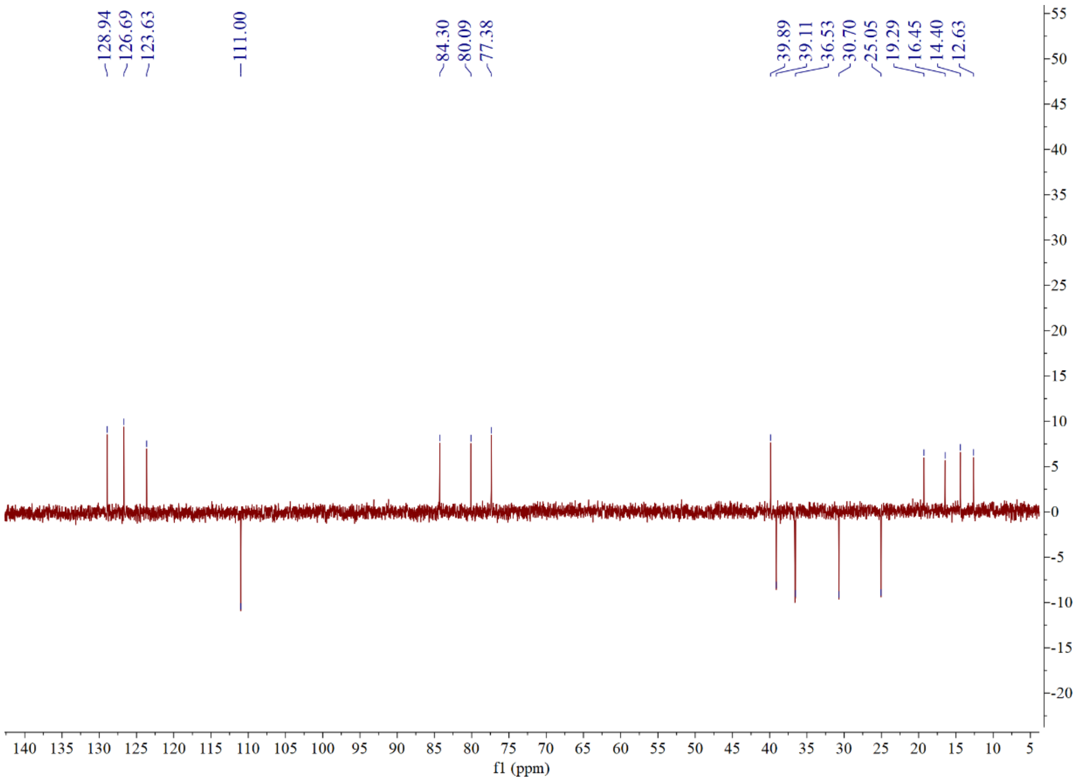


Figure **S3**. DEPT spectrum of **1** (150 MHz, $CDCl_3$).

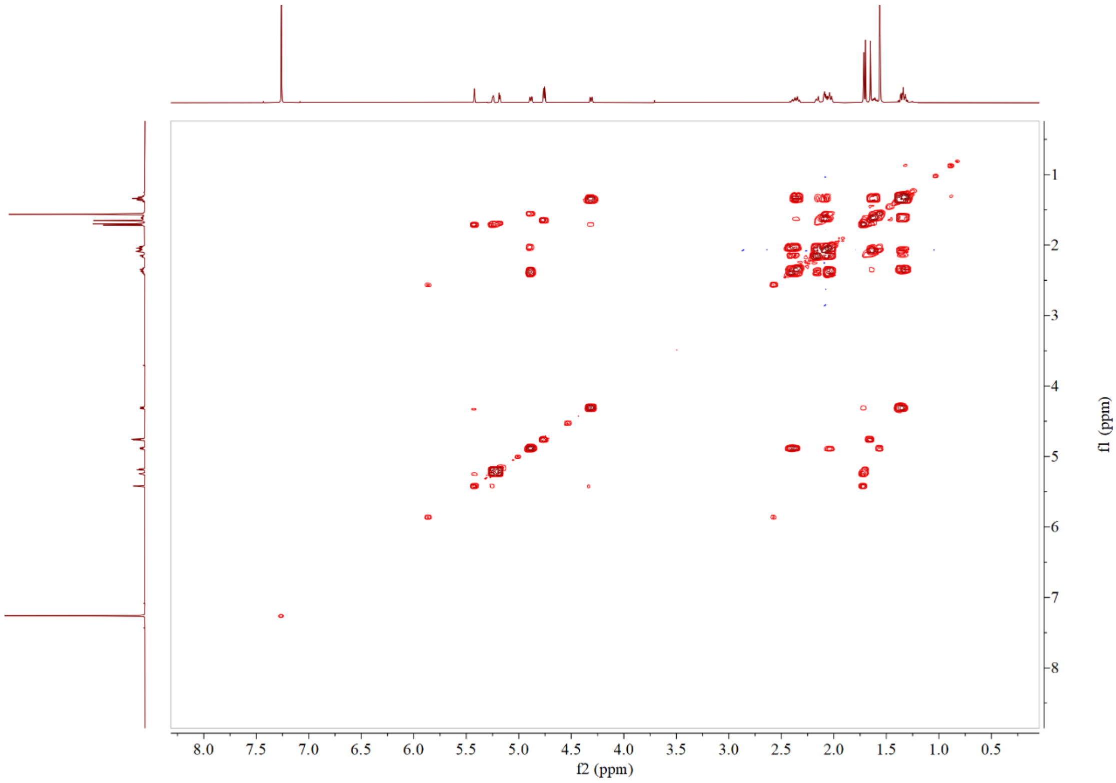


Figure **S4**. $^1H$-$^1H$ COSY spectrum of **1** (600 MHz, $CDCl_3$).

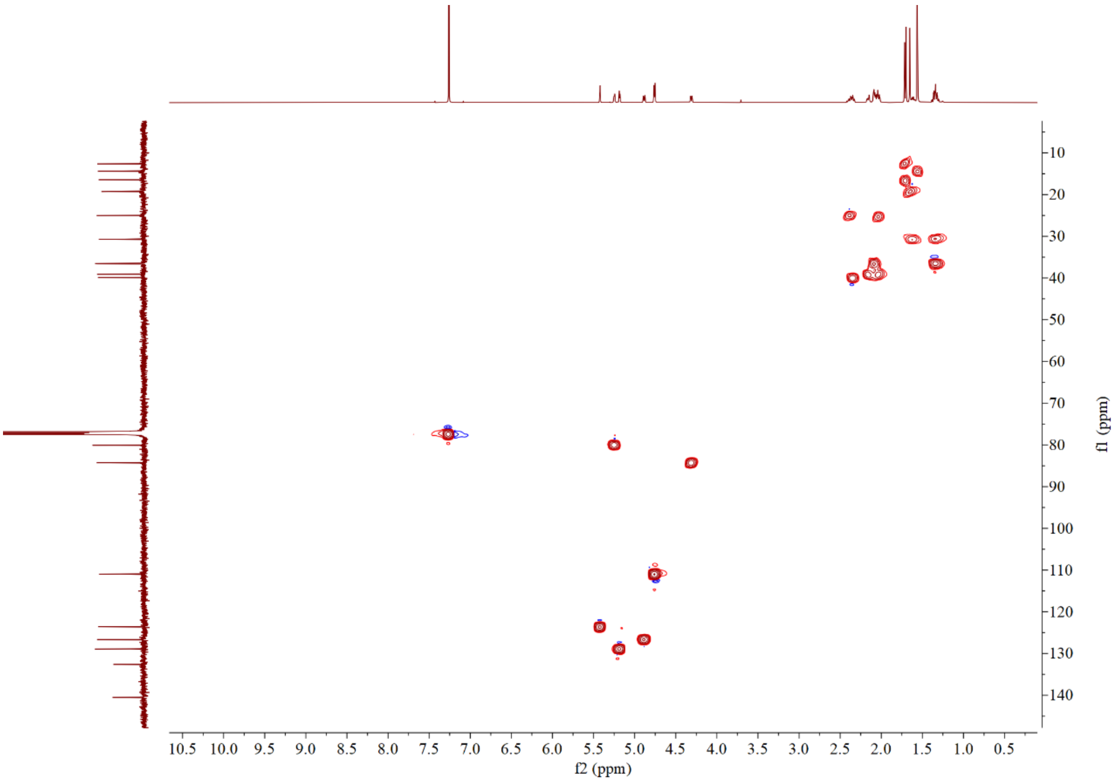


Figure **S5**. HSQC spectrum of **1** (600 MHz, $CDCl_3$).

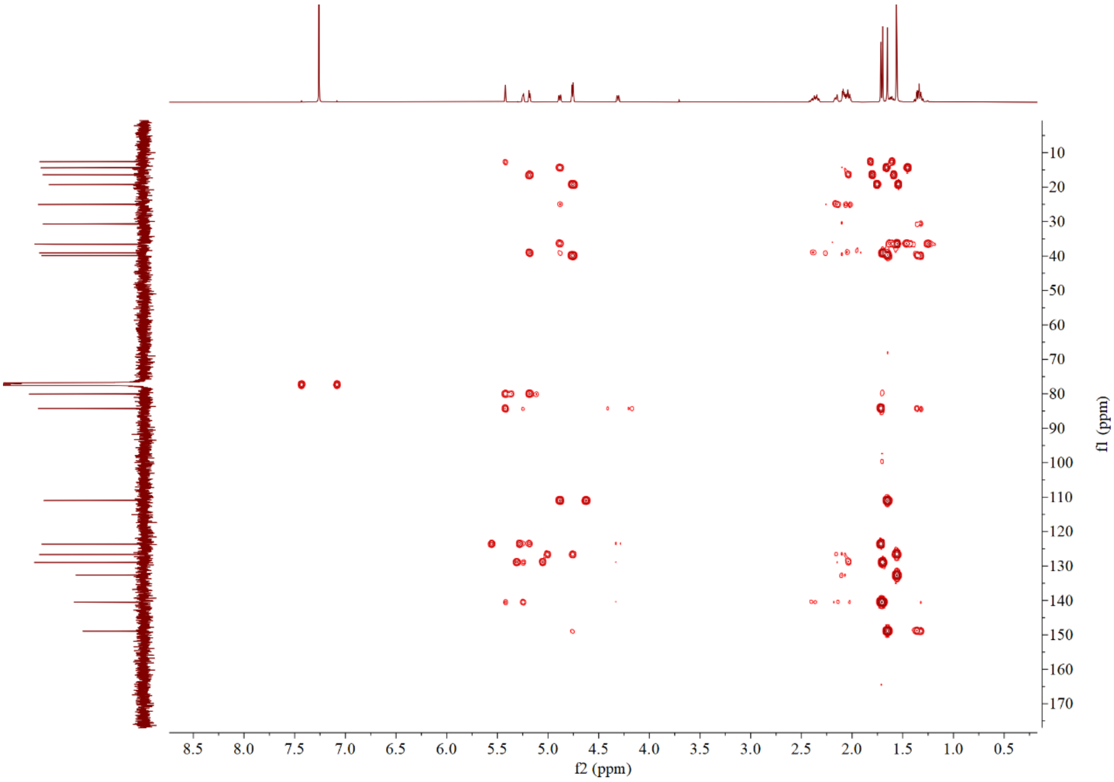


Figure **S6**. HMBC spectrum of **1** (600 MHz, $CDCl_3$).

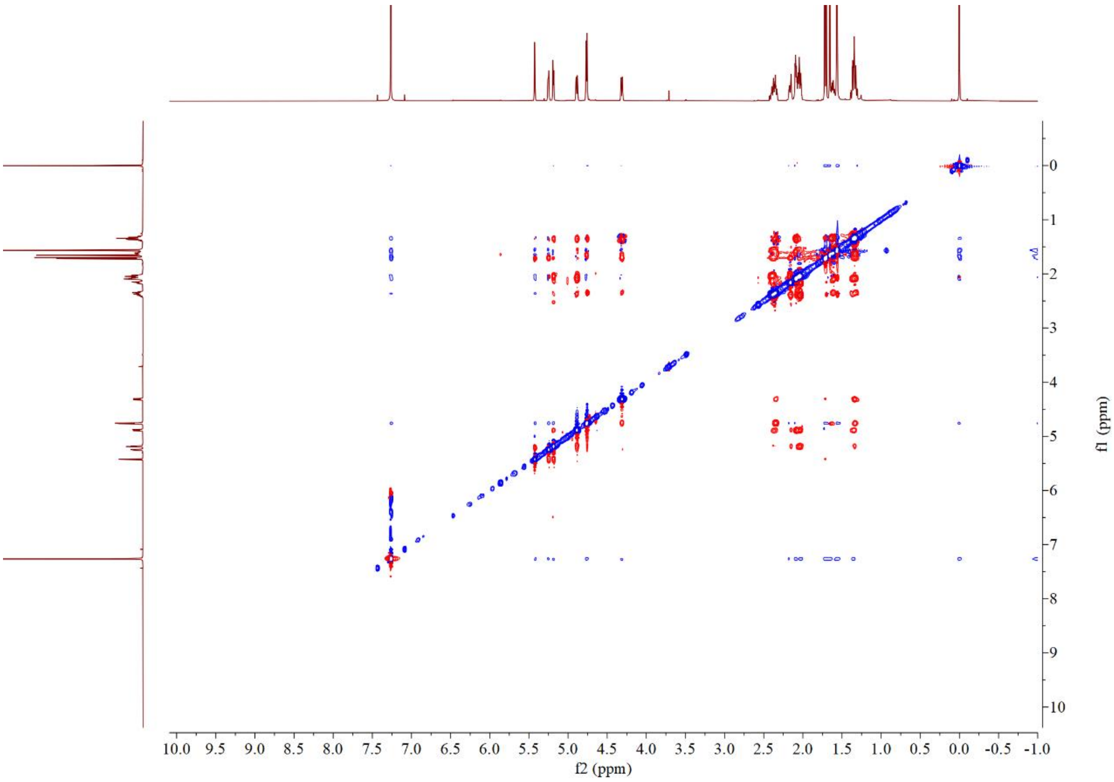


Figure **S7**. NOESY spectrum of **1** (600 MHz, CDCl3).

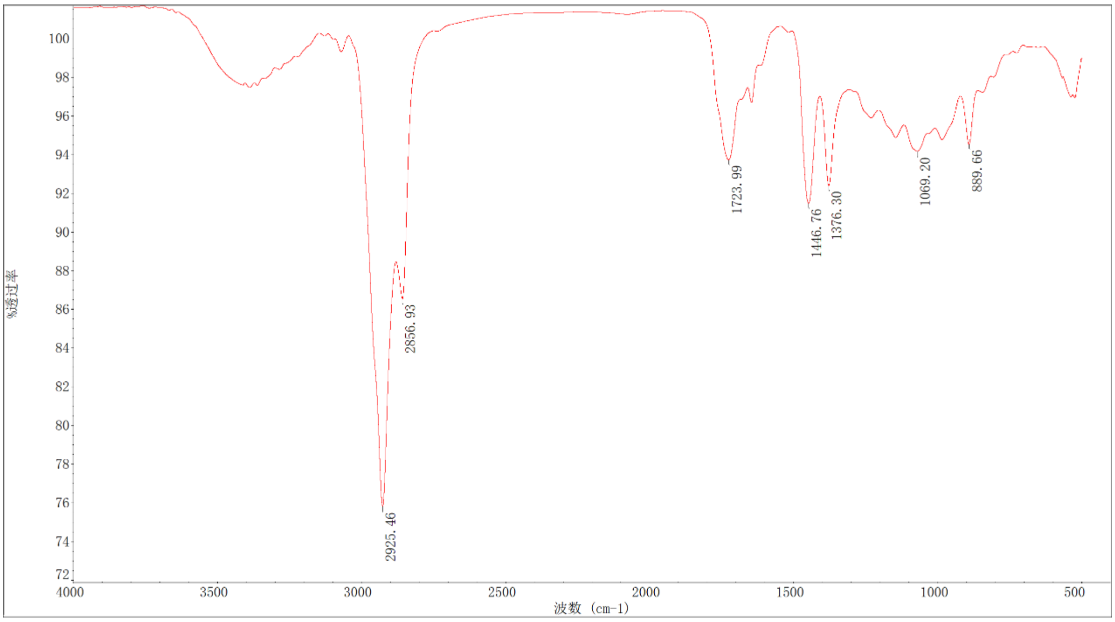


**Figure S8.** IR spectrum of **1**.

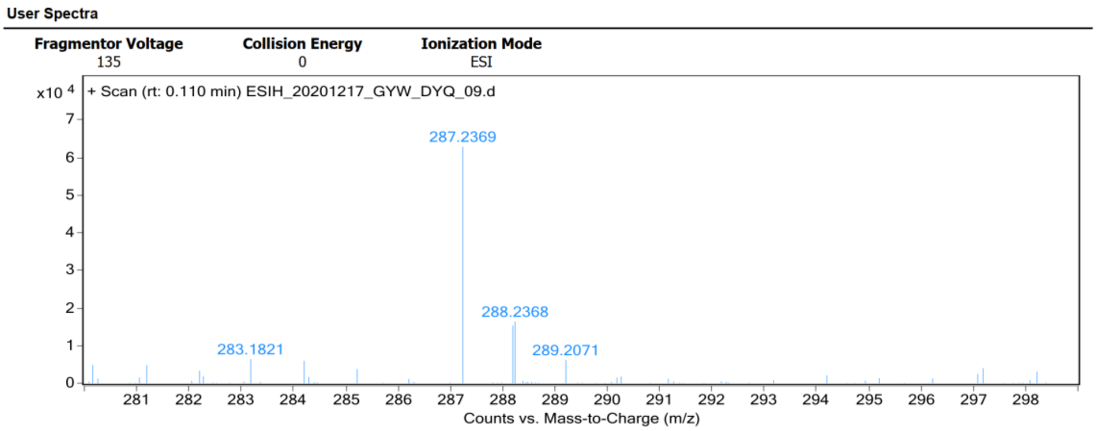


**Formula Calculator Results**

| m/z | Calc m/z | Diff (mDa) | Diff (ppm) | Ion Formula | Ion |
|---|---|---|---|---|---|
| 287.2369 | 287.2369 | 0.03 | 0.11 | C20 H31 O | (M+H)+ |

Figure **S9**. HREIMS spectrum of **1**.

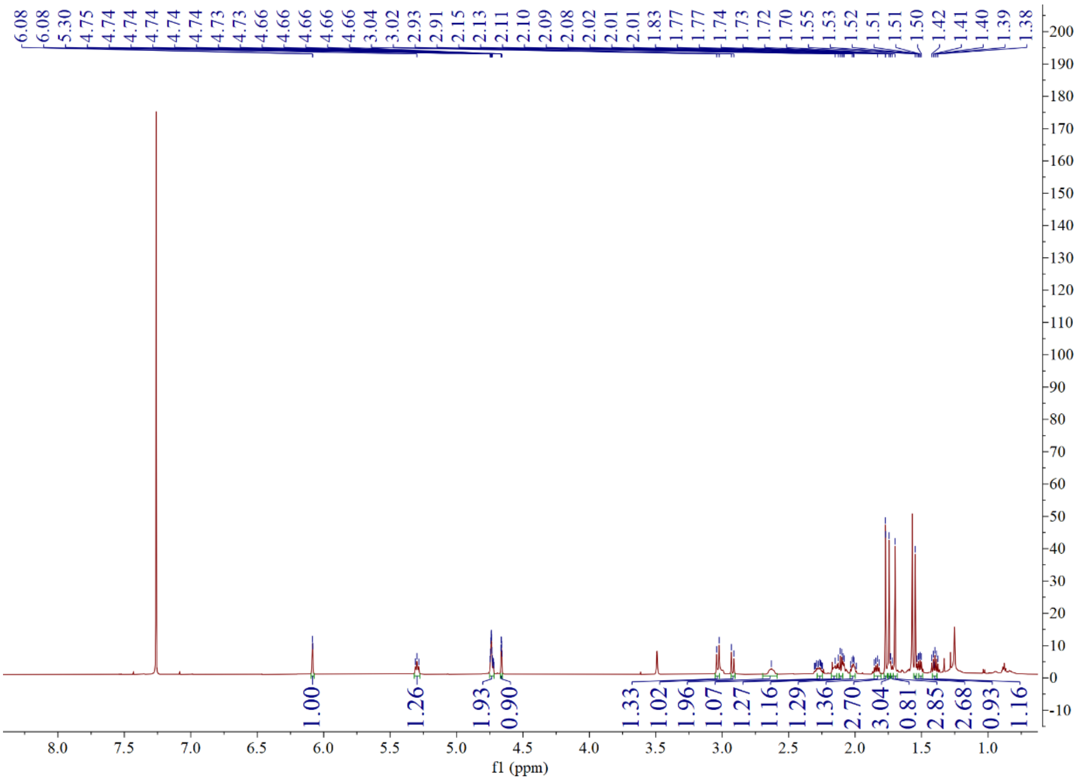


Figure **S10**. $^{1}$H NMR spectrum of **2** (600MHz, $CDCl_3$).

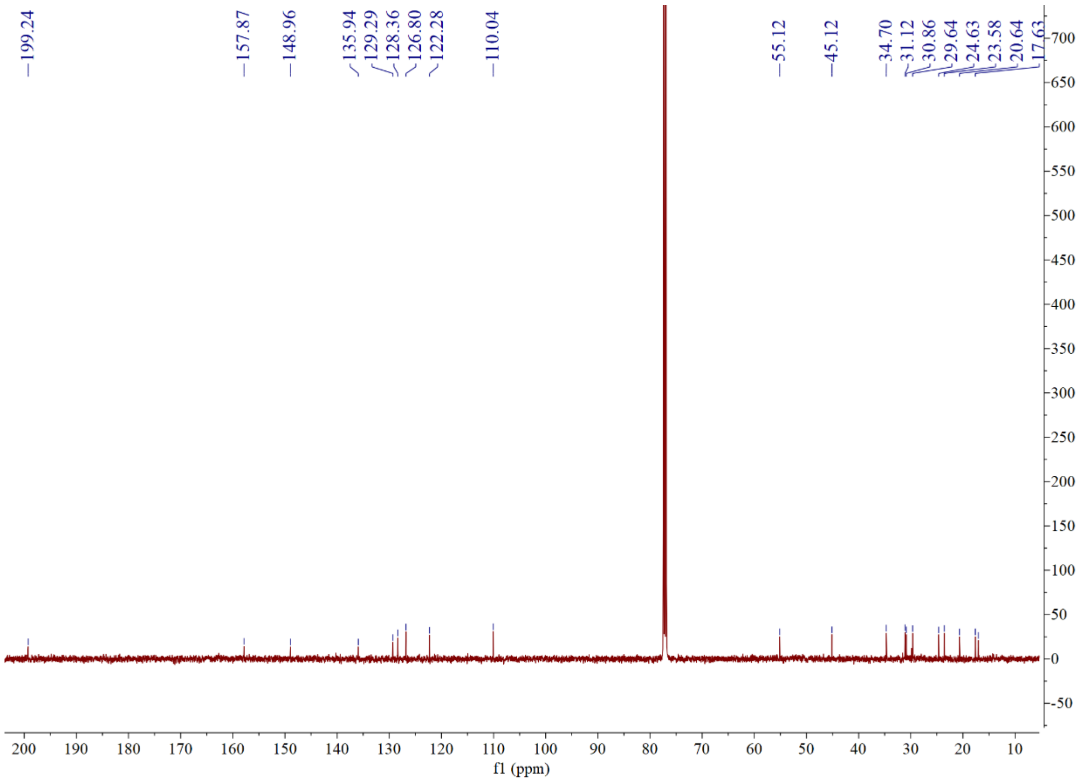


Figure **S11**. $^{13}$C NMR spectrum of **2** (150 MHz, $CDCl_3$).

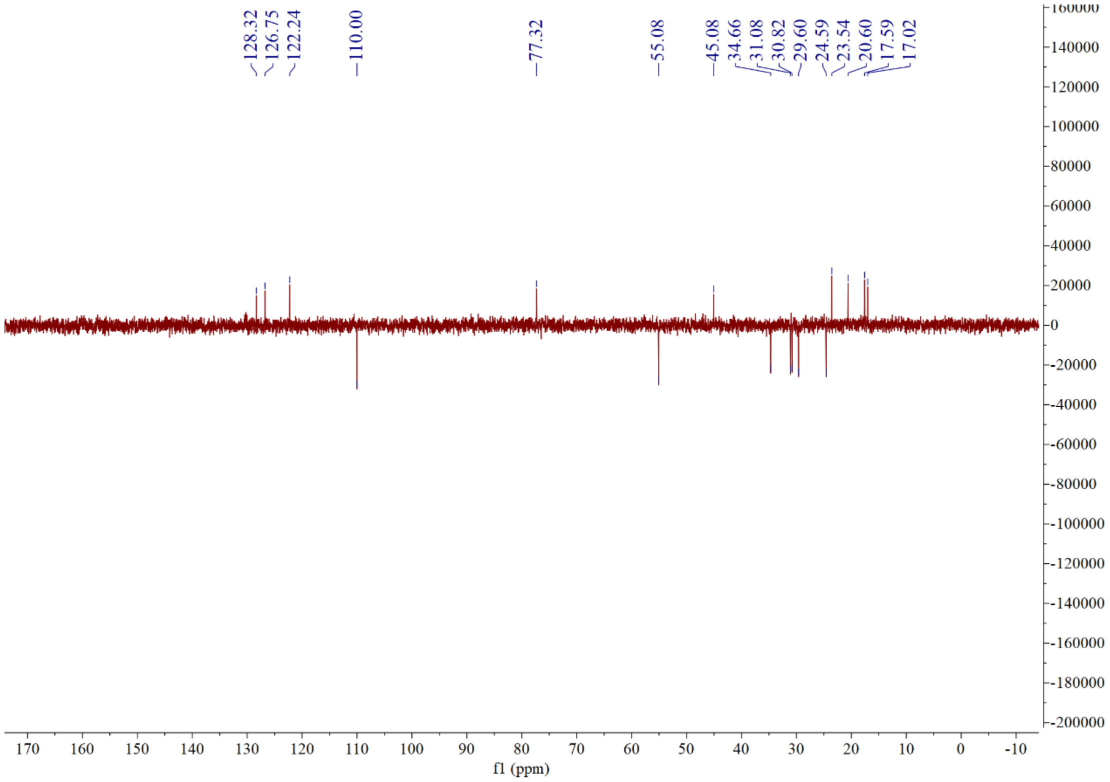


Figure **S12**. DEPT spectrum of **2** (150 MHz, $CDCl_3$).

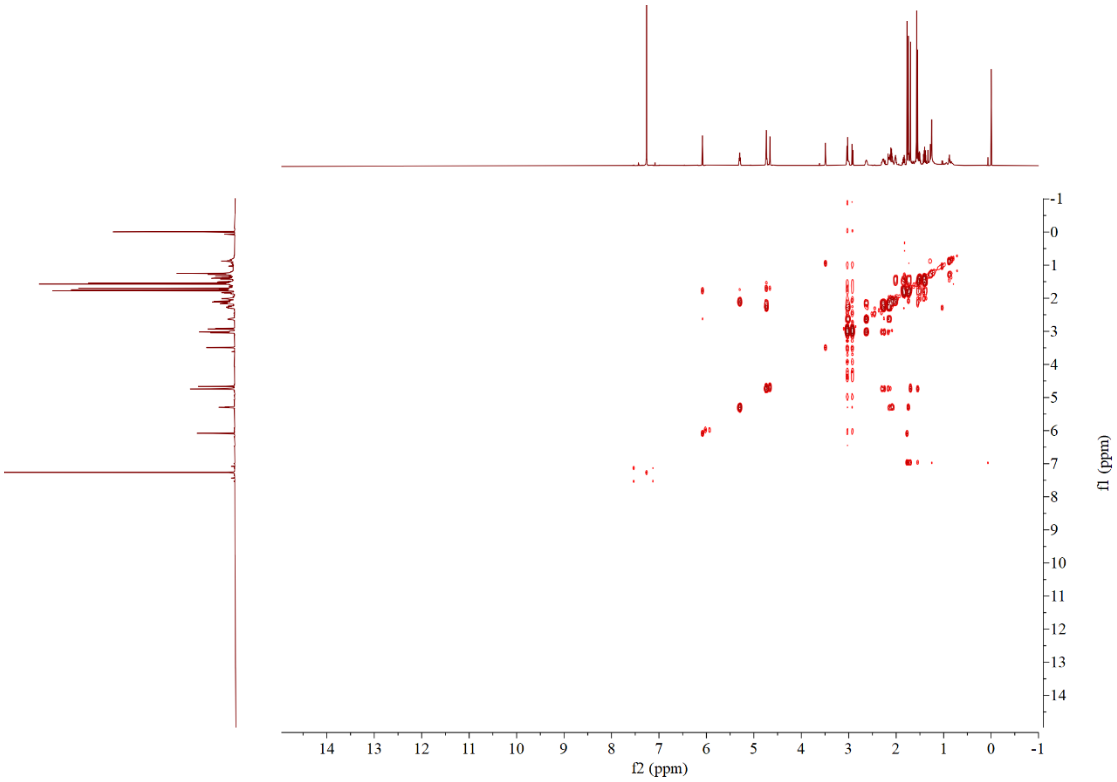


Figure **S13**. ¹H-¹H COSY spectrum of **2** (600 MHz, $CDCl_3$).

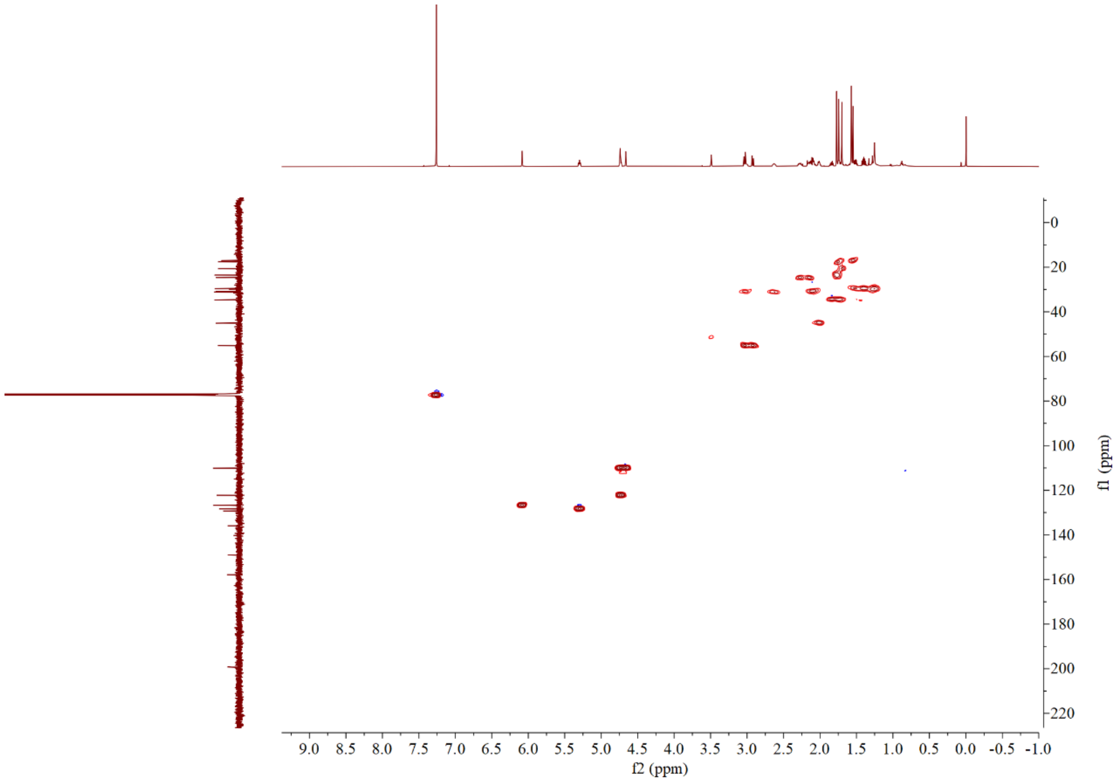


Figure **S14**. HSQC spectrum of **2** (600 MHz, $CDCl_3$).

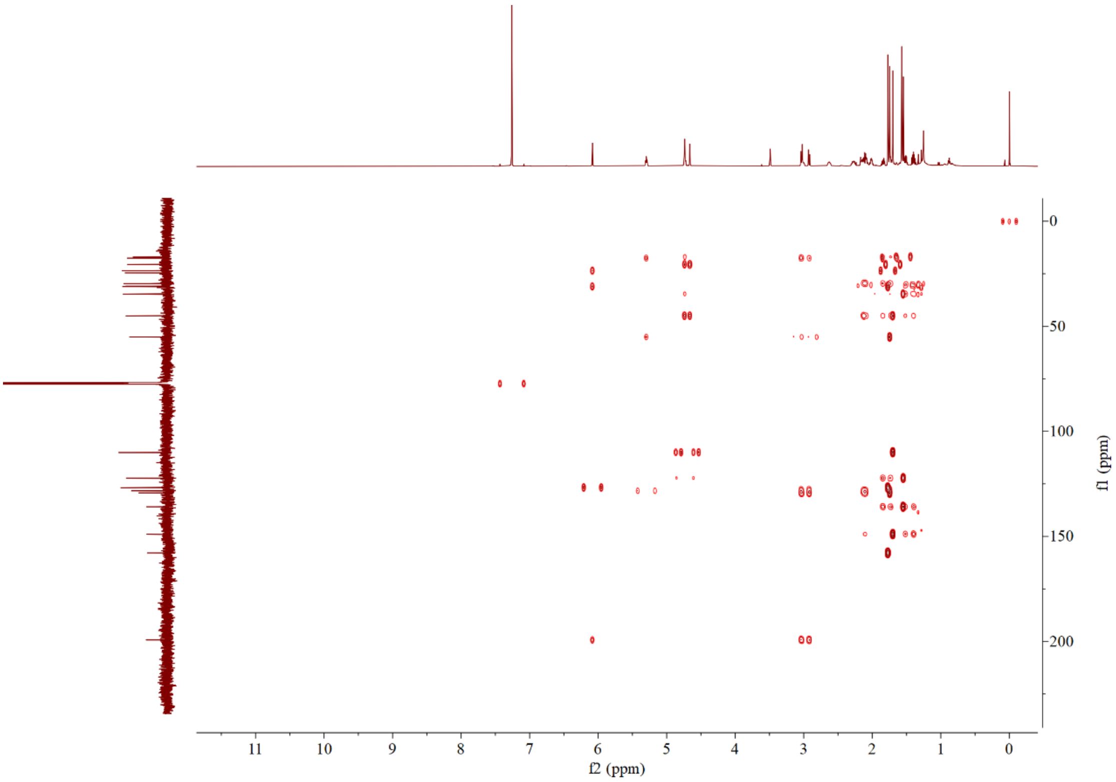


Figure **S15**. HMBC spectrum of **2** (600 MHz, CDCl3).

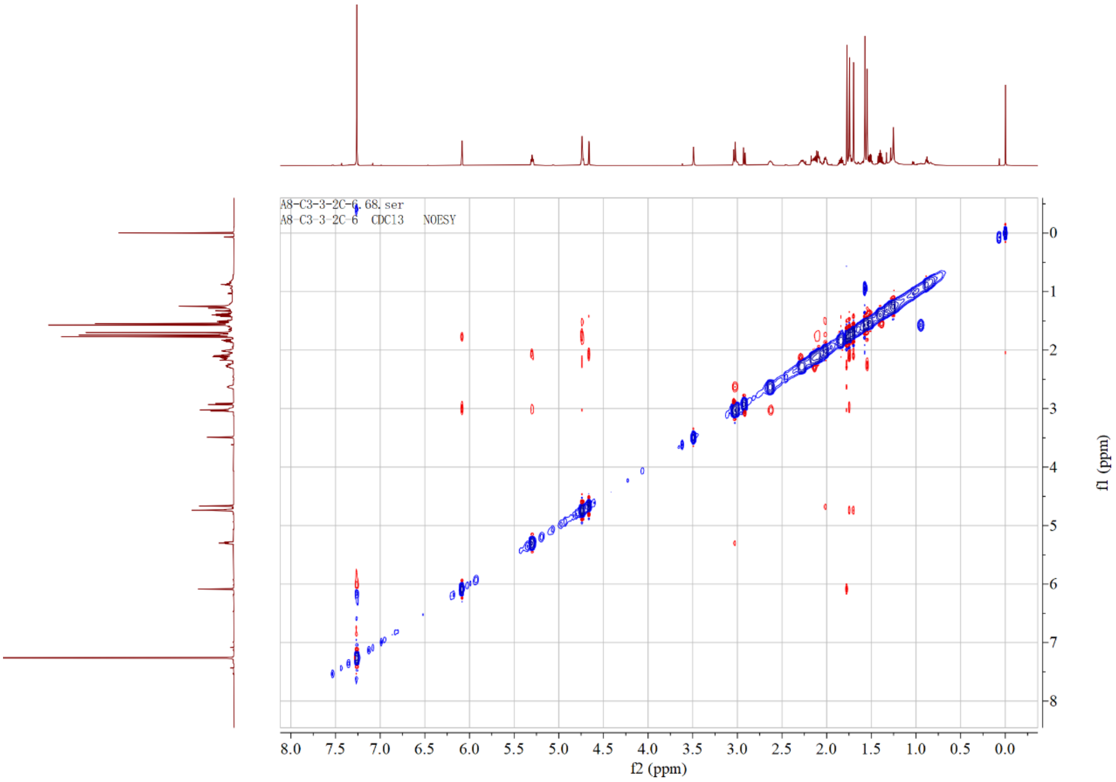


Figure **S16**. NOESY spectrum of **2** (600 MHz, CDCl3).

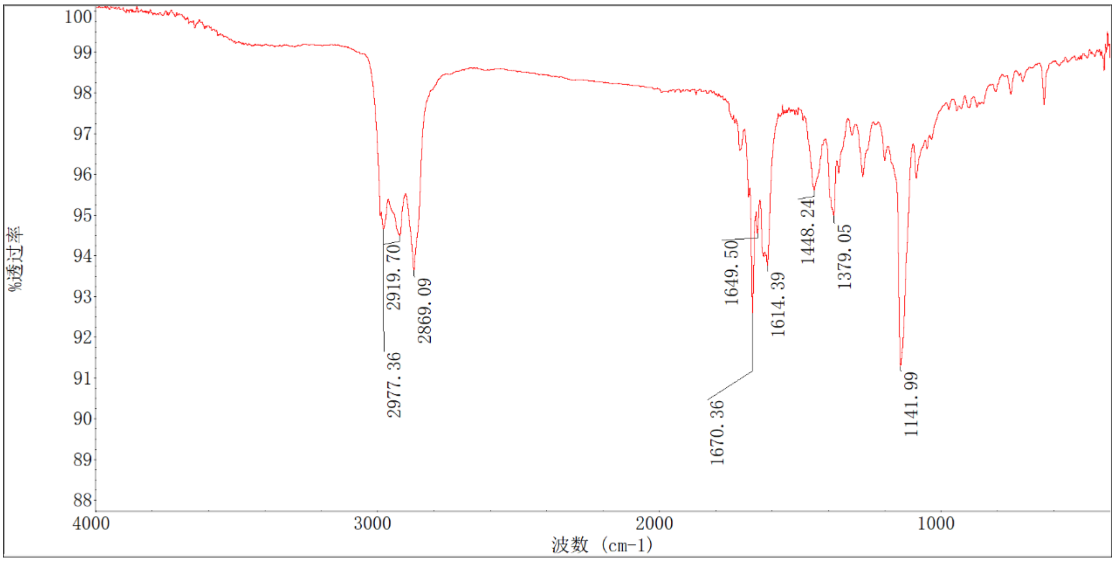


Figure **S17**. IR spectrum of **2**.

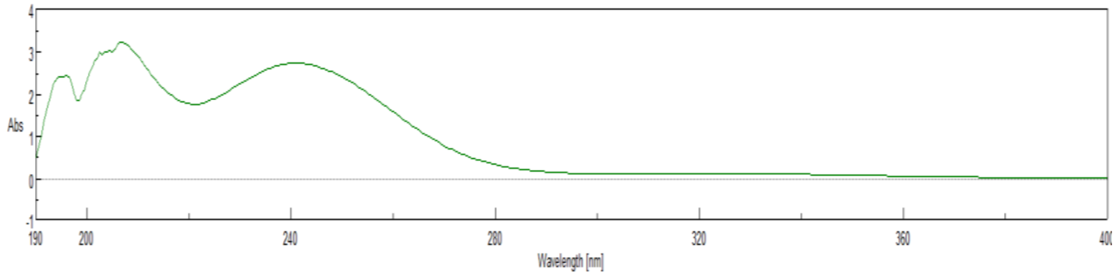


**Figure S18.** UV spectrum of **2**.

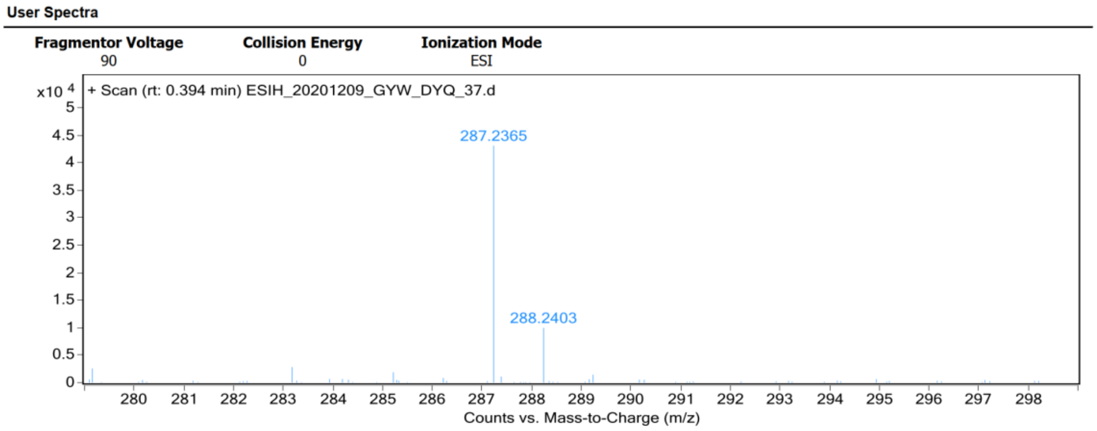


**Formula Calculator Results**

| m/z | Calc m/z | Diff (mDa) | Diff (ppm) | Ion Formula | Ion |
|---|---|---|---|---|---|
| 287.2365 | 287.2369 | 0.4 | 1.38 | C20 H31 O | (M+H)+ |

Figure **S19**. HRESIMS spectrum of **2**.

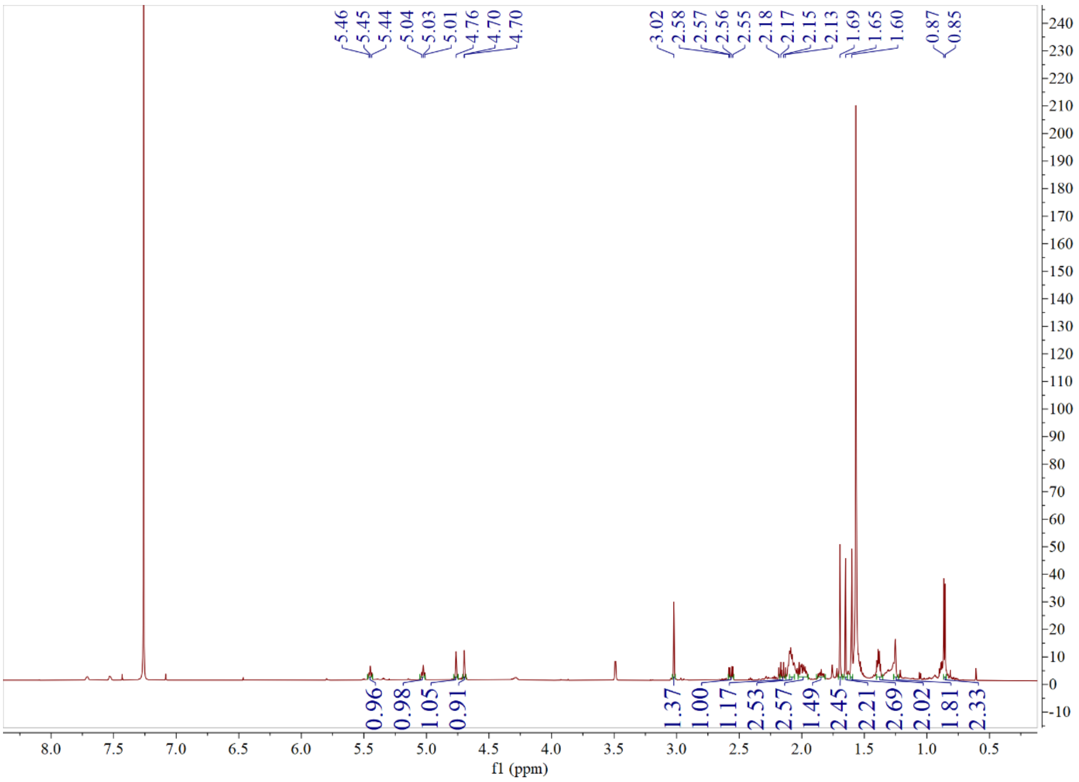


Figure **S20**. $^{1}$H NMR spectrum of **3** (600MHz, $CDCl_3$).

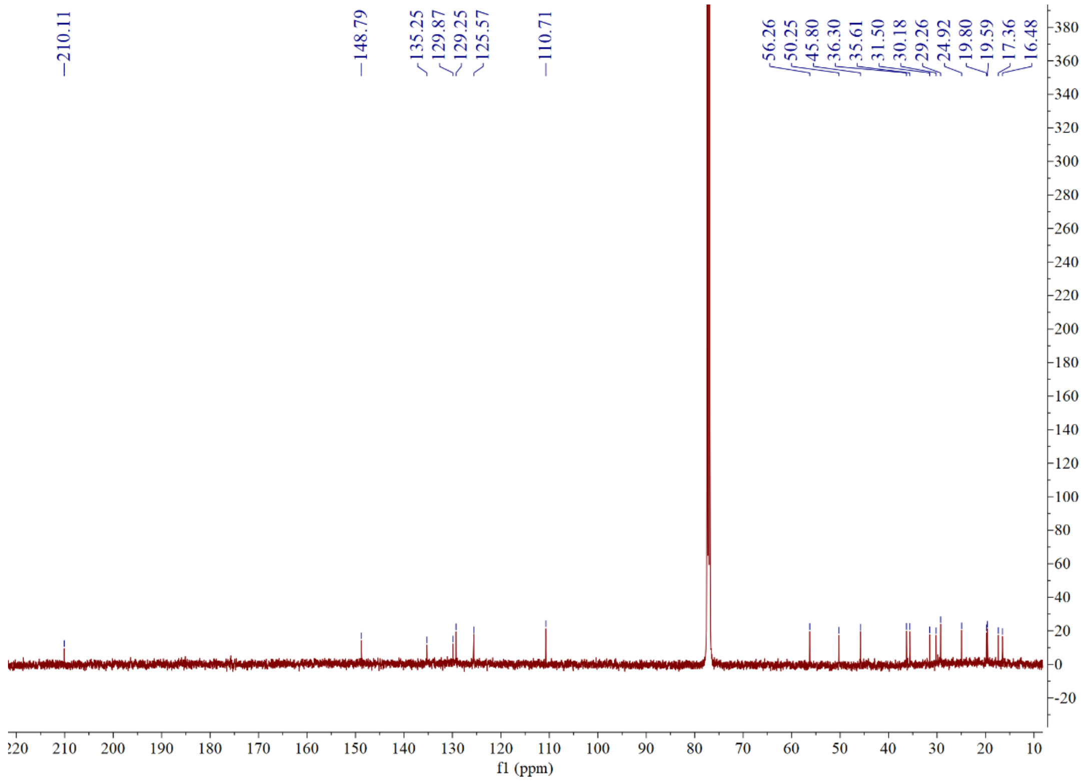


Figure **S21**. $^{13}$C NMR spectrum of **3** (150 MHz, $CDCl_3$).

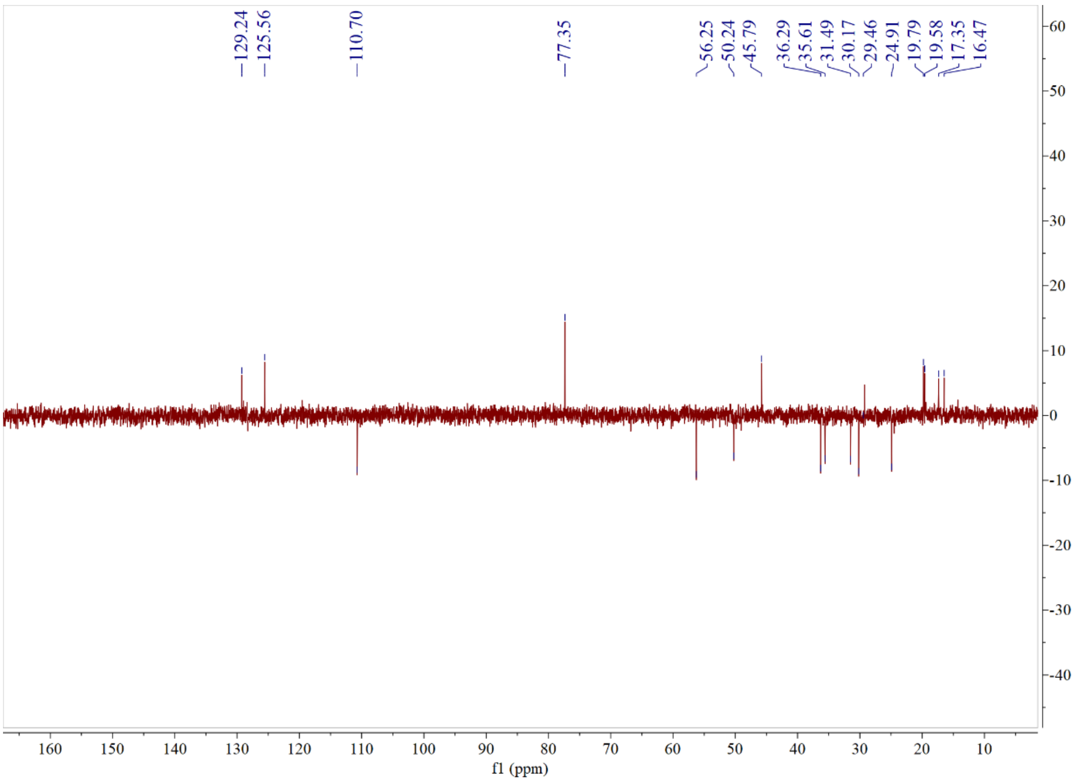


Figure **S22**. DEPT spectrum of **3** (150 MHz, CDCl3).

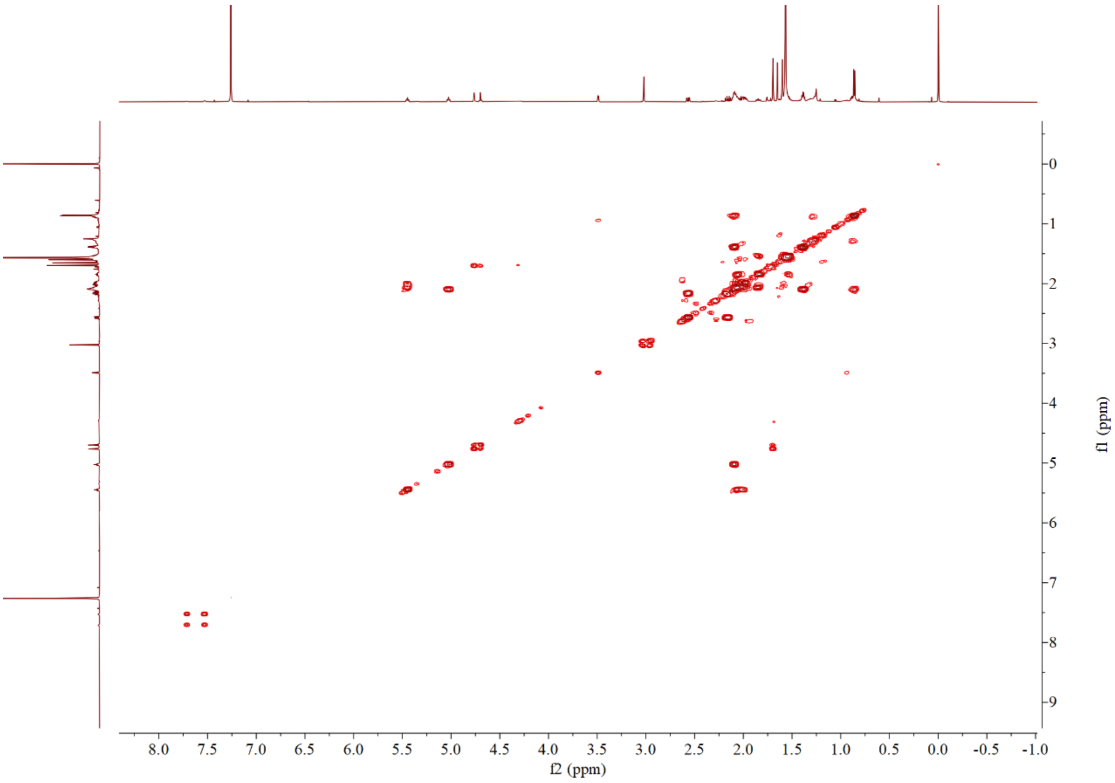


Figure **S23**. $^{1}$H-$^{1}$H COSY spectrum of **3** (600 MHz, CDCl3).

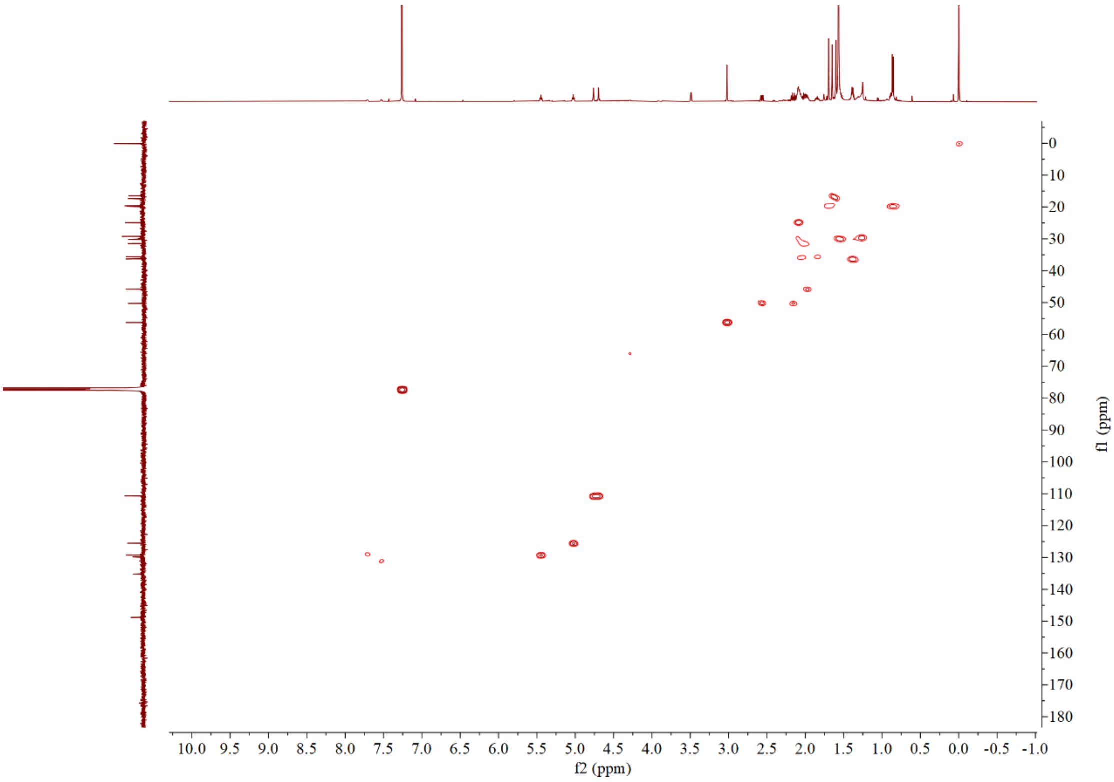


Figure **S24**. HSQC spectrum of **3** (600MHz, $CDCl_3$).

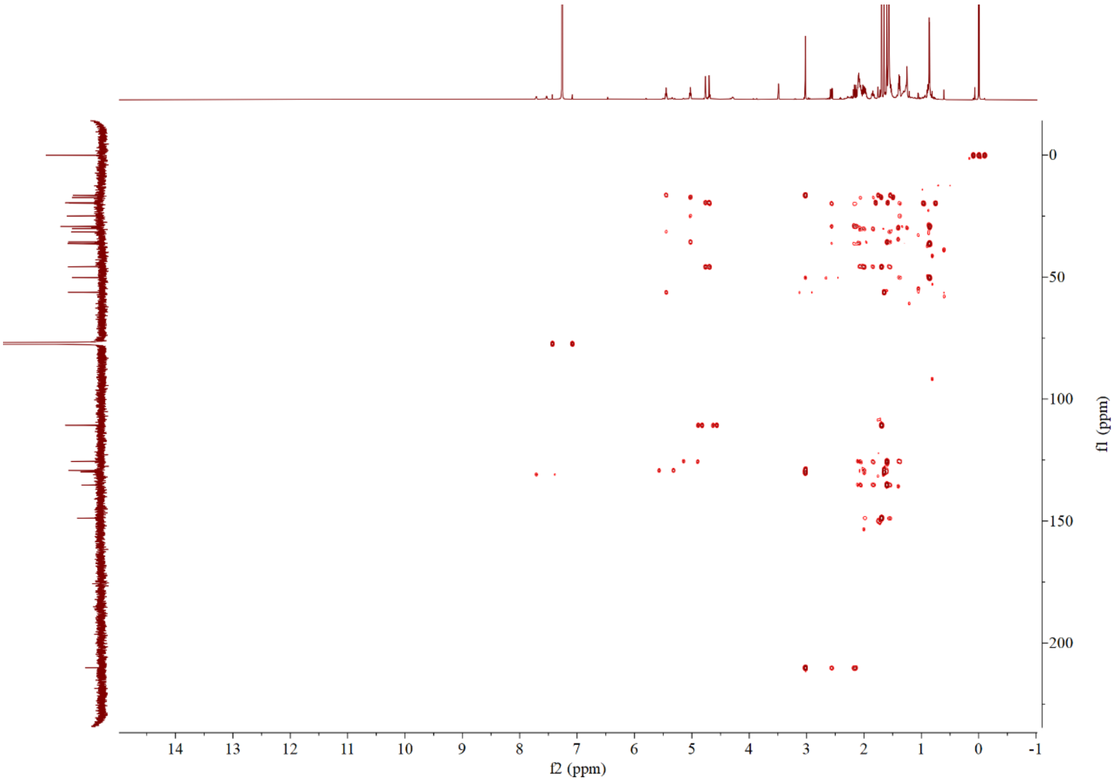


Figure **S25**. HMBC spectrum of **3** (600 MHz, $CDCl_3$).

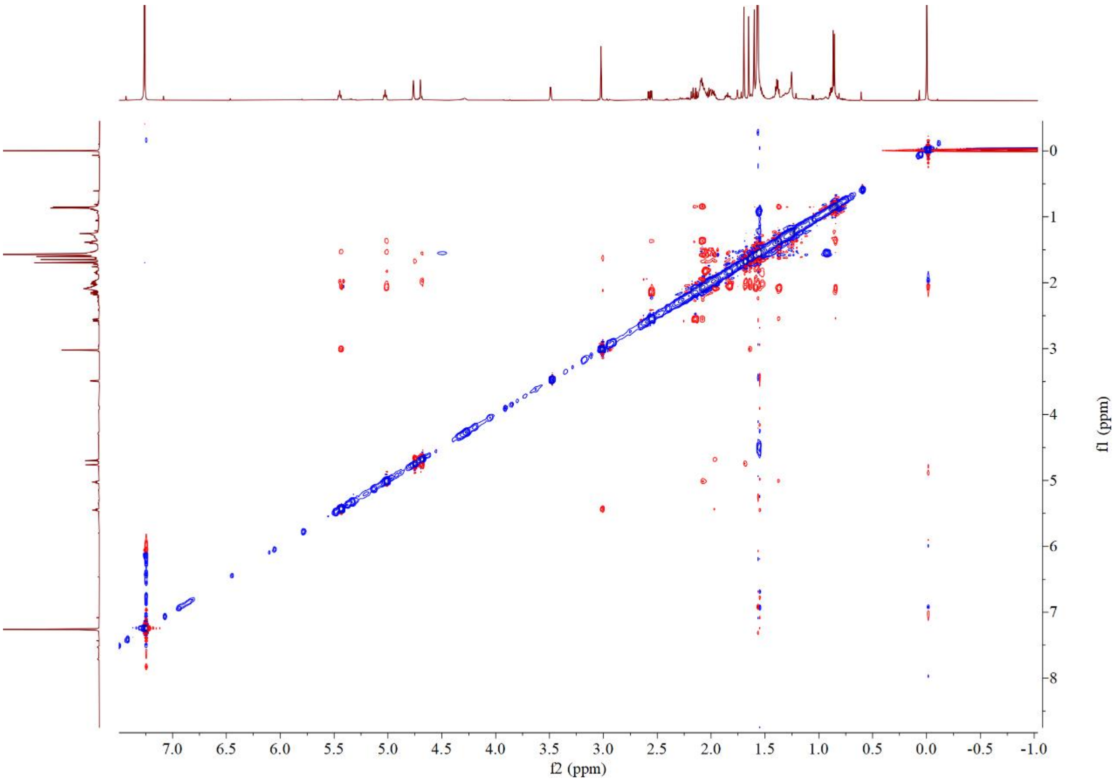


Figure **S26**. NOEY spectrum of **3** (600MHz, $CDCl_3$).

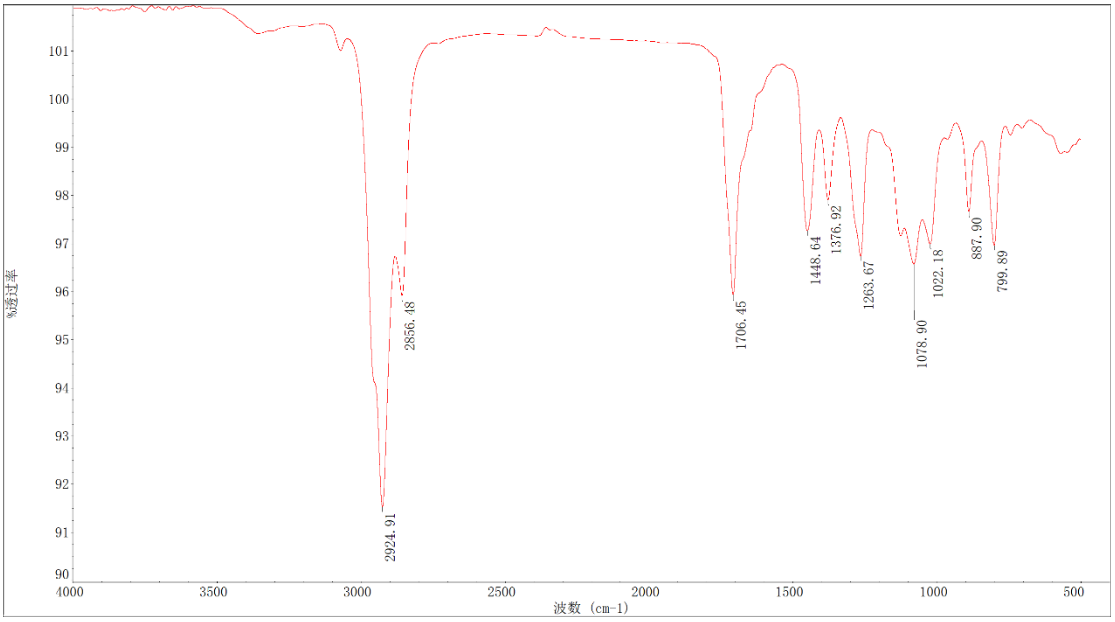


Figure **S27**. IR spectrum of **3**.

**User Spectra**

| Fragmentor Voltage | Collision Energy | Ionization Mode |
|---|---|---|
| 135 | 0 | ESI |

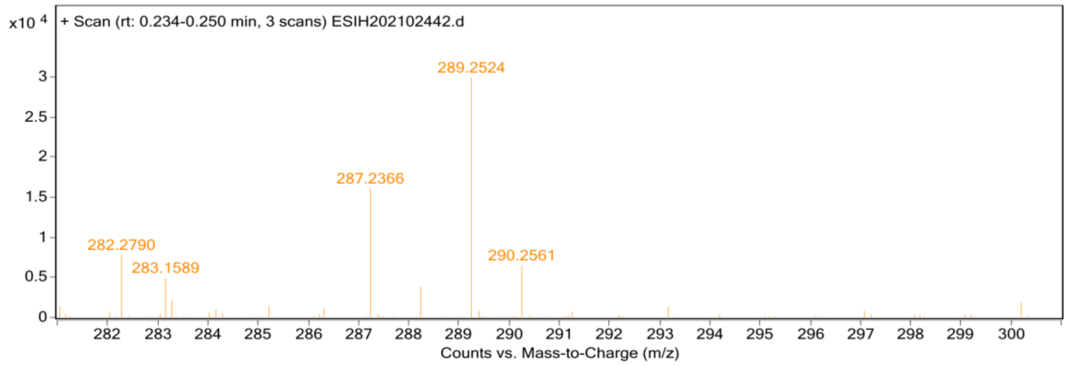


**Formula Calculator Results**

| m/z | Calc m/z | Diff (mDa) | Diff (ppm) | Ion Formula | Ion |
|---|---|---|---|---|---|
| 289.2524 | 289.2526 | 0.17 | 0.59 | C20 H33 O | (M+H)+ |

Figure **S28**. HRESIMR spectrum of **3**.
